# VeloRM: disentangling pre- and post-splicing RNA modification dynamics at single-cell resolution

**DOI:** 10.1093/nar/gkag645

**Published:** 2026-06-30

**Authors:** Haozhe Wang, Bowen Song, Zhixing Wu, Yigan Zhang, Jiayi Li, Yuxin Zhang, Anh Nguyen, Jionglong Su, Daniel J Rigden, Yijun Tang, Guifang Jia, Jia Meng

**Affiliations:** Department of Biosciences and Bioinformatics, Center for Intelligent RNA Therapeutics, Suzhou Key Laboratory of Cancer Biology and Chronic Disease, School of Science, Xi'an Jiaotong-Liverpool University, Suzhou, Jiangsu 215123, China; Institute of Biomedical Research, Regulatory Mechanism and Targeted Therapy for Liver Cancer Shiyan Key Laboratory, Hubei provincial Clinical Research Center for Precise Diagnosis and Treatment of Liver Cancer, Taihe Hospital, Hubei University of Medicine, Shiyan, Hubei 442000, China; Department of Computer Science, University of Liverpool, Liverpool L7 8TX, United Kingdom; Department of Public Health, School of Medicine, Nanjing University of Chinese Medicine, Nanjing, Jiangsu 210023, China; Synthetic and Functional Biomolecules Center, Beijing National Laboratory for Molecular Sciences, Key Laboratory of Bioorganic Chemistry and Molecular Engineering of Ministry of Education, College of Chemistry and Molecular Engineering, Peking University, Beijing 100871, China; Department of Biosciences and Bioinformatics, Center for Intelligent RNA Therapeutics, Suzhou Key Laboratory of Cancer Biology and Chronic Disease, School of Science, Xi'an Jiaotong-Liverpool University, Suzhou, Jiangsu 215123, China; Department of Computer Science, University of Liverpool, Liverpool L7 8TX, United Kingdom; School of AI and Advanced Computing, XJTLU Entrepreneur College, Xi’an Jiaotong-Liverpool University, Suzhou, Jiangsu 215123, China; Institute of Biomedical Research, Regulatory Mechanism and Targeted Therapy for Liver Cancer Shiyan Key Laboratory, Hubei provincial Clinical Research Center for Precise Diagnosis and Treatment of Liver Cancer, Taihe Hospital, Hubei University of Medicine, Shiyan, Hubei 442000, China; Department of Biosciences and Bioinformatics, Center for Intelligent RNA Therapeutics, Suzhou Key Laboratory of Cancer Biology and Chronic Disease, School of Science, Xi'an Jiaotong-Liverpool University, Suzhou, Jiangsu 215123, China; Institute of Systems, Molecular and Integrative Biology, University of Liverpool, Liverpool L7 8TX, United Kingdom; State Key Laboratory of Epigenetic Regulation and Intervention, Institute of Biophysics, Chinese Academy of Sciences, Beijing 100101, China; Department of Computer Science, University of Liverpool, Liverpool L7 8TX, United Kingdom; School of AI and Advanced Computing, XJTLU Entrepreneur College, Xi’an Jiaotong-Liverpool University, Suzhou, Jiangsu 215123, China; Institute of Systems, Molecular and Integrative Biology, University of Liverpool, Liverpool L7 8TX, United Kingdom; Institute of Biomedical Research, Regulatory Mechanism and Targeted Therapy for Liver Cancer Shiyan Key Laboratory, Hubei provincial Clinical Research Center for Precise Diagnosis and Treatment of Liver Cancer, Taihe Hospital, Hubei University of Medicine, Shiyan, Hubei 442000, China; Synthetic and Functional Biomolecules Center, Beijing National Laboratory for Molecular Sciences, Key Laboratory of Bioorganic Chemistry and Molecular Engineering of Ministry of Education, College of Chemistry and Molecular Engineering, Peking University, Beijing 100871, China; Department of Biosciences and Bioinformatics, Center for Intelligent RNA Therapeutics, Suzhou Key Laboratory of Cancer Biology and Chronic Disease, School of Science, Xi'an Jiaotong-Liverpool University, Suzhou, Jiangsu 215123, China; Institute of Biomedical Research, Regulatory Mechanism and Targeted Therapy for Liver Cancer Shiyan Key Laboratory, Hubei provincial Clinical Research Center for Precise Diagnosis and Treatment of Liver Cancer, Taihe Hospital, Hubei University of Medicine, Shiyan, Hubei 442000, China; Institute of Systems, Molecular and Integrative Biology, University of Liverpool, Liverpool L7 8TX, United Kingdom

## Abstract

RNA modifications critically regulate RNA function and fate, yet their dynamic changes across the RNA life cycle and during cellular transitions remain largely unexplored. Here we introduce VeloRM, a computational framework that captures RNA modification dynamics at single-cell resolution. VeloRM uniquely disentangles presplicing and postsplicing epitranscriptomes, enabling for the first time the identification and differentiation of modification sites on transcripts before versus after splicing. By modeling the velocity of epitranscriptomic changes, VeloRM predicts future RNA modification states and reconstructs cellular trajectories directly from epitranscriptome information. Application to single-cell datasets profiling m6A and A-to-I editing demonstrates that VeloRM accurately recapitulates known trajectory patterns in cell cycle and differentiation. Notably, VeloRM reveals for the first time a set of m6A sites that are hyper-methylated on prespliced RNAs near splice junctions, with dynamic patterns that unveil clear functional implications in splicing regulation. VeloRM represents a rigorous yet powerful tool that opens unprecedented opportunities to study epitranscriptome dynamics during biological transitions.

## Graphical Abstract

**Graphical Abstract:**
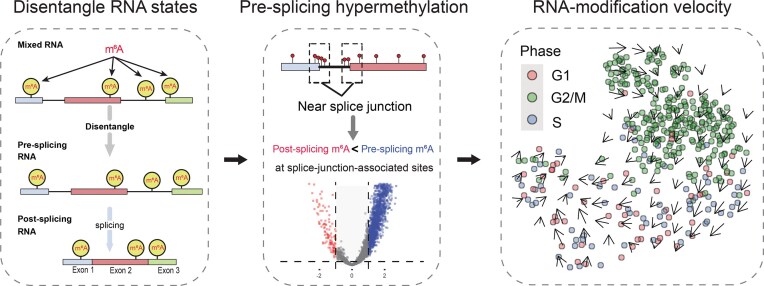

## Introduction

Recent breakthroughs in RNA epigenetics [1, 2] and epitranscriptomics [3] have demonstrated that reversible RNA modifications serve as critical regulators of gene expression [4]. With over 330 naturally existing chemical modifications identified across all domains of life [5], these epigenetic marks influence virtually every aspect of RNA biology—from stability [6] and subcellular localization [7] to processing [8] and translational control [9]. Their dysregulation has been increasingly linked to numerous disease states [10–13], particularly cancer pathogenesis [14–16]. Beyond canonical RNA metabolism, these modifications have emerged as key regulators of chromatin architecture [17, 18], splicing fidelity [19], viral and transposable element activity [20, 21], positioning them as promising therapeutic targets.

Advances in high-throughput sequencing [22, 23] and chemical labeling technologies [8, 24] have substantially deepened our understanding of the distribution, dynamics, and functional relevance of RNA modifications [7, 25]. Antibody-based MeRIP-seq or m^6^A-seq [26, 27] first enabled transcriptome-wide m^6^A profiling, revealing millions of conserved sites across species [28, 29]. Complementary approaches like RNA bisulfite sequencing [30] provided single-base resolution for m^5^C mapping through selective cytosine deamination [31, 32]. The advent of nanopore direct RNA sequencing (DRS) [33–36] represents a quantum leap, enabling real-time detection of diverse modifications including m^6^A [37], ψ [37], A-to-I editing [38], polyA tails [39], and m^1^ψ [40] on individual RNA molecules.

More recently, the rapid development of single-cell technologies [41] has transformed molecular biology by enabling the simultaneous profiling of genomic, transcriptomic, proteomic, epigenomic, and metabolomic features in thousands of individual cells [42]. In the field of RNA modifications, these advances have facilitated the emergence of powerful single-cell epitranscriptomic methods that allow detection and quantification of modifications such as N6-methyladenosine (m^6^A) with unprecedented cellular resolution [43]. Notably, scDART-seq [44] represents the first single-cell method for high-throughput profiling of m^6^A across thousands of cells. It employs a fusion protein comprising the m^6^A-binding YTH domain and the cytidine deaminase APOBEC1 to induce C-to-U editing at cytidine residues adjacent to m^6^A sites, as guided by the DRACH consensus motif (D = A/G/U, R = A/G, H = A/C/U) [26, 27]. In parallel, other approaches such as m^6^A-SAC-seq [45], PicoMeRIP-seq [46], sn-m^6^A-CT [47], and scm^6^A-seq [48] have extended single-cell m^6^A profiling to low-input, nuclear, and developmental contexts, collectively. These methods often co-capture full transcriptomic profiles alongside epitranscriptomic signals, enabling multilayered analysis of gene expression and modification status within the same cells. When combined with droplet-based platforms such as 10x Genomics or SMART-seq2 [49], they provide high-throughput capabilities suitable for trajectory inference and subpopulation discovery. Beyond sequencing, imaging-based techniques such as m^6^A-ISH-PLA [50] and single-molecule microscopy [51] further allow spatially resolved visualization of m^6^A at subcellular resolution.

While technological innovations have greatly enhanced our ability to study m^6^A at the single-cell level, they have also highlighted longstanding biological questions. One central issue concerns the timing of m^6^A deposition, which remains unclear [52]. Some studies have suggested co-transcriptional installation [53, 54], while others have proposed post-transcriptional modification mediated by the exon-junction complex [55–57]. This ongoing uncertainty highlights the critical need to directly and dynamically capture RNA modification kinetics. Recent evidence further refines this understanding by demonstrating that the cytoplasmic degradation of modified RNAs—rather than active regulation by m^6^A erasers—plays a primary role in determining m^6^A levels [58]. Through the integration of transcriptome-wide data and mechanistic modeling, this study provides strong support for the idea that RNA metabolism is a dominant force shaping m^6^A patterns. These findings not only consolidate prior observations but also underscore the importance of rigorous experimental and analytical strategies when evaluating m^6^A dynamics.

In parallel, the development of RNA velocity has revolutionized single-cell transcriptomics by enabling the inference of future gene expression states through quantifying splicing kinetics from presplicing sequencing reads and postsplicing ones [59]. Originally introduced by previous work [59, 60], this core idea has become instrumental for reconstructing developmental trajectories [61], mapping lineage relationships [62], and capturing transcriptional dynamics across heterogeneous cell populations [63]. More recent extensions have incorporated aspects such as RNA degradation rates and stochasticity [61, 64], multilineage system [64], and the velocity of protein [65] and chromatin modification [66]. However, to the best of our knowledge, the concept of RNA velocity has not yet been rigorously extended to RNA modifications.

Building on these advances and motivated by emerging questions in the epitranscriptomics field, we propose a conceptual extension of RNA velocity—epitranscriptome velocity—to characterize the temporal dynamics of transcriptome RNA modifications, such as, m^6^A or A-to-I editing. This approach is designed to explore how dynamic changes in the epitranscriptome with respect to splicing process at the single-cell level. Unlike conventional RNA velocity, which infers gene expression trends from transcript abundance, epitranscriptome velocity focuses on tracking the gain and loss of specific RNA modifications over (pseudo) time. This paradigm shift—from expression to chemical modification—offers a complementary framework for understanding RNA fate, revealing epitranscriptome regulatory layers that extend beyond the direct transcriptional control. To translate this conceptual framework into a practical tool, we developed VeloRM, which effectively untangles the precursor and mature epitranscriptomes and enables rigorous quantification of RNA modification velocity in single cells at single site resolution, facilitating the prediction of their future cell states in a dynamic process.

## Methods

### Annotation of reads derived from presplicing and postsplicing RNAs

Due to the low sequencing depth of 10x Genomics data [49], single-cell datasets exhibited high sparsity, particularly for epitranscriptomic analyses. We therefore utilized SMART-seq2 [67] data, a read-based technology, to enable precise annotation of presplicing and postsplicing reads at single-read resolution. This annotation framework is broadly applicable to next-generation sequencing (NGS) datasets, including scRNA-seq [68], DART-seq [69], scDART-seq [44], and MeRIP-seq [26], with SMART-seq2 [49] serving as a representative high-coverage platform.

In NGS data, the presence of multiple isoforms per gene complicates read assignment, as reads may align to regions shared among transcripts. To resolve this ambiguity, we obtained exon and intron annotation BED files from the UCSC Genome Browser and noted overlapping intervals between exonic and intronic regions. Using BEDTools [70], we generated nonoverlapping genomic partitions by: (i) pure intronic regions (present in all transcripts) by subtracting exonic coordinates from intronic regions; (ii) pure exonic regions (present in all transcripts) by subtracting intronic coordinates from exonic regions. This generated nonoverlapping genomic partitions for unambiguous feature classification.

The following classification rules were applied to annotate reads as postsplicing, presplicing, or ambiguous:

1. A read is classified as postsplicing if it does not overlap any intronic region, overlaps pure exonic regions, and contains a splice junction; presplicing or ambiguous reads containing splice junctions are also reassigned to this category.
2. A read is annotated as presplicing if it overlaps pure intronic regions.
3. A read is annotated as ambiguous if it overlaps intronic regions but not pure intronic regions (excluded from downstream analyses).

For DRS data, reads were classified based on splicing status. Reads spanning splice junctions were annotated as postsplicing RNAs, whereas reads lacking splice junctions and overlapping regions consistently annotated as intronic across all transcript isoforms were classified as presplicing RNAs.

### Extraction of m^6^A signals from DART-seq, scDART-seq, and DRS

Raw sequencing data were obtained from the GEO database (DART-seq: GSE125780 [69], GSE297551 [71]; scDART-seq: GSE180954 [44]) and aligned to the human hg38 reference genome using STAR [72]. or DRS (GSE132971) [73], raw reads were basecalled using Guppy [74], and aligned to GRCh38 using Minimap2 [75].

Following read annotation, reads were separated into presplicing and postsplicing groups, and two corresponding BAM files were generated for each dataset and processed independently.

In DART-based systems, m^6^A-modified cytosines are chemically converted to uracils and detected as C-to-T mutations in sequencing data. Candidate sites were identified by detecting C-to-T mutations within AC motifs using Samtools [76] with default parameters. For each site, mutated (m^6^A) and unmutated reads were quantified using the GenomicAlignments R package [77].

For DART-seq, two independent datasets were analyzed. GSE125780 (three replicates: three experimental and three YTH-mutant controls) yielded 38 899 candidate sites, while GSE297551 (11 replicates: 11 experimental and 11 controls) yielded 5 954 045 candidate sites. Methylation status was assessed using DESeq2 [78] separately for presplicing and postsplicing RNAs, each compared to the corresponding control group. Sites with log₂FC > 1 were defined as putative m^6^A-modified sites.

For scDART-seq, 1599 cells (1209 test and 390 YTH domain-mutated negative controls) yielded 2371 331 candidate sites. We calculated the error rate for each site as the proportion of reads that were neither C nor T (i.e. nonconversion events) among all reads covering A, C, T, and G, and excluded sites with an error rate exceeding 1%, retaining 450 974 candidates. We then applied the SigRM [79] to evaluate the methylation status of these 450 974 sites at the single-cell level, separately for presplicing and postsplicing RNA, each compared to the corresponding presplicing or postsplicing RNA from control cells. Sites with a median *P*-value below 0.05 under each condition were considered as putative m^6^A-modified sites for that specific condition.

For DRS, m^6^A sites were identified using m^6^Anet [80], retaining sites with a modification probability ≥0.9. This yielded 383 sites in presplicing RNAs and 13 018 sites in postsplicing RNAs.

To quantify the DRS read-level imbalance, classifiable reads were counted after presplicing and postsplicing annotation. This yielded 1 235 694 presplicing reads and 3 733 708 postsplicing reads, corresponding to 24.9% and 75.1% of classifiable reads, respectively. The total exon-aligned read length was 3.76-fold higher for postsplicing than presplicing reads.

### Comparison of the presplicing and postsplicing m^6^A epitranscriptomes

For DART-seq, we retained sites with an error rate ≤1%. This yielded 284 sites from 38 899 candidates in GSE125780 and 41 279 sites from 5954 045 candidates in GSE297551 for downstream analysis. Differential methylation between presplicing and postsplicing RNAs was assessed using DESeq2 [78] and a paired binomial test based on YTH samples only. Sites with an absolute log₂ fold change (|log₂FC|) > 1 and *P* ≤.05 were defined as differentially methylated.

For scDART-seq, sites with insufficient read support (median coverage <10 in either presplicing or postsplicing RNA) were excluded from the initial 450 974 candidates, yielding a high-confidence set of 37 199 sites. Differential methylation was evaluated using both DESeq2 [78] and a paired binomial test based on matched presplicing and postsplicing measurements within the same cells. Sites satisfying |log₂FC| > 1 and *P* ≤.05 in each method were considered differentially methylated.

For DRS, 235 m^6^A sites were shared between presplicing and postsplicing RNAs. Comparison of methylation levels at these sites revealed higher m^6^A levels in presplicing RNAs than in postsplicing RNAs.

### Characterization of transient m^6^A dynamics during cell cycle progression

We developed an integrated computational framework to systematically dissect transient m^6^A methylation dynamics at single-cell resolution by combining cell cycle analysis with kinetic modeling. Cell cycle phase annotations (G1: 55 cells; S: 66 cells; G2/M: 267 cells) were assigned based on canonical marker gene expression, as described in [79].

We applied the VeloRM framework to 37 199 high-confidence m^6^A sites, of which 36 739 had reads in both postsplicing/presplicing and methylated/unmethylated conditions. After filtering out sites with low postsplicing–presplicing read correlation (Spearman ≥ 0.05) and low slope (≥0.05) in both conditions, 34 410 sites remained. Among these, 196 sites were associated with marker genes, and we focused on 142 high-confidence sites showing strong methylation signals (meta log_2_ odds ratio ≥1 in both presplicing and postsplicing transcripts).

We projected current methylation states (log_2_ odds ratios) of the 142 postsplicing sites into low-dimensional space using UMAP [81] and *t*-Distributed Stochastic Neighbor Embedding (*t*-SNE) [82]. To capture dynamic changes, VeloRM simultaneously modeled methylated and unmethylated RNA populations, integrating them to infer methylation transitions based on log₂ odds ratios. Correlation matrices between predicted future and current methylation states were computed and converted into a probabilistic transition matrix that reflects the likelihood of state changes. This matrix allowed for the visualization of both global methylation dynamics and individual cell trajectories. Finally, stationary probabilities derived from the transition matrix were used to identify attractor states, reflecting stable m^6^A modification patterns throughout cell cycle progression. These probabilities were normalized to a 0–1 scale and interpreted as pseudo-time, with lower values indicating early stages and higher values corresponding to later stages in the cycle.

### A-to-I RNA editing site detection from single cell RNA-seq data

We obtained raw sequencing data (GSE99933 and GSE151334) from the GEO database [83] and aligned the reads to the mouse mm39 reference genome using STAR [72]. After read annotation, the processed reads were separated into distinct BAM files for presplicing and postsplicing RNA for independent analysis. A-to-I RNA editing events were detected as A-to-G mismatches in the sequencing data, reflecting the conversion of adenosine to inosine by ADAR enzymes and subsequent interpretation as guanosine during reverse transcription [84]. Candidate editing sites were identified by detecting A-to-G variants in the aligned BAM files using Samtools [76] with default parameters. We then quantified editing levels using the GenomicAlignments [77] R package by counting reads supporting the edited (G) versus reference (A) alleles at each candidate site.

Chromaffin cell differentiation dataset: From the initial analysis of 384 cells, we identified 33 550 potential editing sites. After applying stringent quality filters that excluded sites with error rates exceeding 1%, we retained 1019 high-confidence A-to-I editing sites.

Multicell-type broad RNA spectrum dataset: From the initial analysis of 2153 cells, we identified 59 349 potential editing sites. After applying stringent quality filters that excluded sites with error rates exceeding 1%, we retained 1931 high-confidence A-to-I editing sites.

### Comparison of the presplicing and postsplicing A-to-I editome

Differential editing between presplicing and postsplicing RNAs was assessed using both DESeq2 [78] and a paired binomial test based on matched measurements within the same cells. Sites with an absolute log₂ fold change (|log₂FC|) > 1 and *p* ≤.05 were defined as differentially edited.

### Characterization of transient A-to-I RNA editing dynamics during cell differentiation progression

#### Chromaffin cell differentiation dataset

Cell differentiation phase annotations and gene expression embeddings were obtained from [83]. VeloRM was applied to 1019 high-confidence sites, of which 189 retained reads across both postplicing/presplicing and modified/unmodified conditions. Filtering for sufficient correlation (Spearman ≥ 0.05) and minimum slope (≥0.05) in both conditions retained 52 sites. Sites with detectable editing in ≥ 10 cells (Beta-value > 0) were further selected, yielding 36 sites.

Temporal dynamics were modeled with VeloRM by integrating modified and unmodified RNA populations to infer editing transitions based on M-values. Correlation matrices between predicted future and current states were transformed into a probabilistic transition matrix, enabling visualization of both global A-to-I editing dynamics and individual cell trajectories along differentiation.

#### Multicell-type broad RNA spectrum dataset

Cell differentiation phase annotations and gene expression embeddings were obtained from [85] for 1123 cells. VeloRM was applied to 1 931 high-confidence sites, of which 217 retained reads across both postplicing/presplicing and modified/unmodified conditions. Filtering for correlation (Spearman ≥ 0.05) and slope (≥0.05) retained 41 sites. Sites with detectable editing in ≥3 cells (beta-value > 0) were selected, yielding 27 sites.

Temporal dynamics were inferred using VeloRM on modified and unmodified populations, integrating beta-values to estimate editing transitions. Correlation matrices were transformed into probabilistic transition matrices to visualize global editing dynamics and individual cell trajectories across differentiation.

### Theory and computational methods

Glossary of notation:

-
$U/S$ – presplicing (*U*)/postsplicing (*S*) RNA
-
$m/w$ – modified (*m*)/unmodified (*w*) reads
-
$i/j$ – site index (*i*)/cell index (*j*)
-
$u/s$ – size-normalized presplicing (*u*)/postsplicing (*s*) RNA

### Velocity model

Let $u_{ij}^m$ and $u_{ij}^w$ denote the size-normalized methylated and nonmethylated read counts of presplicing RNA (presplicing RNA), and ${\mathrm{\ }}s_{ij}^m{\rm}$ and $s_{ij}^w$ denote the size-normalized methylated and nonmethylated read counts of postsplicing RNA (postsplicing RNA) for the ${{i}^{th}}$ site and the ${{j}^{th}}$ cell. Additionally, ${{u}_{ij}}$ and ${{s}_{ij}}$ represent the sum of the size-normalized methylated and nonmethylated read counts for presplicing RNA and postsplicing RNA.


(1) \begin{eqnarray*}
{u_{ij}^m = \frac{{U_{ij}^m}}{{\mathop \sum \nolimits_i U_{ij}^m + \mathop \sum \nolimits_i U_{ij}^w}} = \frac{{U_{ij}^m}}{{\mathop \sum \nolimits_i {{U}_{ij}}}}}
\end{eqnarray*}

(2) \begin{eqnarray*}
{u_{ij}^w = \frac{{U_{ij}^w}}{{\mathop \sum \nolimits_i U_{ij}^m + \mathop \sum \nolimits_i U_{ij}^w}} = \frac{{U_{ij}^w}}{{\mathop \sum \nolimits_i {{U}_{ij}}}}}
\end{eqnarray*}

(3) \begin{eqnarray*}
{s_{ij}^m = \frac{{S_{ij}^m}}{{\mathop \sum \nolimits_i S_{ij}^m + \mathop \sum \nolimits_i S_{ij}^w}} = \frac{{S_{ij}^m}}{{\mathop \sum \nolimits_i {{S}_{ij}}}}}
\end{eqnarray*}

(4) \begin{eqnarray*}
{s_{ij}^w = \frac{{S_{ij}^w}}{{\mathop \sum \nolimits_i S_{ij}^m + \mathop \sum \nolimits_i S_{ij}^w}} = \frac{{S_{ij}^w}}{{\mathop \sum \nolimits_i {{S}_{ij}}}}}
\end{eqnarray*}

(5) \begin{eqnarray*}
{{{u}_{ij}} = u_{ij}^m + u_{ij}^w}
\end{eqnarray*}

(6) \begin{eqnarray*}
{{{s}_{ij}} = s_{ij}^m + s_{ij}^w}
\end{eqnarray*}

Here, $U_{ij}^m$and $U_{ij}^w$ denote the methylated and nonmethylated read counts of presplicing RNA (presplicing RNA), and $S_{ij}^m$and $S_{ij}^w$ denote the methylated and nonmethylated read counts of postsplicing RNA (postsplicing RNA) for the ${{i}^{th}}$ site and the ${{j}^{th}}$ cell.

Building upon established modeling frameworks [59], we formulate a dynamic system to characterize the quantitative relationships between methylated/nonmethylated pre-splicing and post-splicing RNA populations. Focusing specifically on the methylated RNA as a representative case, we model the temporal dynamics of methylated presplicing RNA through a differential equation accounting for two key biological processes: transcriptional production and splicing-mediated depletion. The rate of change in methylated presplicing RNA concentration is given by:


(7) \begin{eqnarray*}
{\frac{{\mathrm{ d}u_i^m}}{{\mathrm{ d}t}} = \alpha _i^m - {{\beta }^m}u_i^m(t)}
\end{eqnarray*}

Here, $\alpha _i^m$ denotes the transcription rate of methylated transcript, which is a constant, and ${{\beta }^m}$ denote the splicing rate of methylated presplicing RNA.

The temporal dynamics of methylated postsplicing RNA are governed by two competing processes: production through splicing of presplicing RNA and degradation of postsplicing RNA. This relationship is described by the differential equation:


(8) \begin{eqnarray*}
{\frac{{\mathrm{ d}s_i^m}}{{\mathrm{ d}t}} = {{\beta }^m}u_i^m(t) - \gamma _i^ms_i^m(t)}
\end{eqnarray*}

The degradation rate $\gamma _i^m$ is the key parameter to be estimated (see *Supplementary Note: Theoretical framework and computational details—Parameter estimation* for implementation procedures and further details).

### Predictive modeling of postsplicing RNA dynamics

After estimating the degradation rate but given the challenges of directly determining transcription rates $\alpha$, we developed two alternative approaches for predicting postsplicing RNA read counts that circumvent this limitation. The following description uses methylated RNA as an example, but the same logic applies to nonmethylated RNA.

### Model I. Constant velocity assumption

This model assumes temporal invariance in the net splicing flux $( {\frac{{\mathrm{ d}s_i^m}}{{\mathrm{ d}t}} = v_i^m{\mathrm{\ is\ a\ constant}}} )$, yielding a linear prediction framework:


(9) \begin{eqnarray*}
{v_i^m = {{\beta }^m}u_i^m(t) - \gamma _i^ms_i^m(t) - o_i^m}
\end{eqnarray*}

(10) \begin{eqnarray*}
{v_{ij}^m = {{\beta }^m}u_{ij}^m - \gamma _i^ms_{ij}^m - o_i^m}
\end{eqnarray*}

(11) \begin{eqnarray*}
s_{ij}^m(t) = {\mathrm{max}}\left( {0,s_{ij}^m + v_{ij}^mt} \right)
\end{eqnarray*}

Here $s_{ij}^m( t )$ is the predicted size-normalized methylated postsplicing RNA read count for the ${{i}^{th}}$ site and ${{j}^{th}}$ cell.

### Model II. Constant presplicing RNA assumption

This alternative model assumes quasi-stationary presplicing RNA levels $[ {\frac{{\mathrm{ d}s_i^m}}{{\mathrm{ d}t}} = {{\beta }^m}u_i^m( 0 ) - \gamma _i^ms_i^m( t )} ]$, resulting in an exponential solution:


(12) \begin{eqnarray*}
s_{ij}^m(t) = \frac{{\ {{\beta }^m}\hat{u}_{ij}^m}}{{\gamma _i^m}}\left( {1 - {{e}^{ - \gamma _i^mt}}} \right) + s_{ij}^m{{e}^{ - \gamma _i^mt}}
\end{eqnarray*}

Here $\ \hat{u}_{ij}^m = {\mathrm{max}}( {0,u_{ij}^m - o_i^m} )$ represents baseline-corrected presplicing count.

### Visualization of cell velocity

To characterize cellular trajectories based on predicted methylation patterns in postsplicing RNA, we employ a dimensionality reduction approach coupled with methylation-aware projections. To visualize the predicted postsplicing RNA metrics of methylation levels, we use the current metrics as the baseline plot. Principal component analysis (PCA), UMAP [81], and *t*-SNE [82] employed techniques for this purpose. These methods enable us to visualize the methylation level metrics at the cellular level.

### Predict methylation level of postsplicing RNAs

We can predict the methylated and nonmethylated read counts of postsplicing RNA based on the previous predictions, as we have learned the degradation rates $\gamma _i^m$ and $\gamma _i^w$ for the methylated and nonmethylated conditions. Let $s_{ij}^m( t )$ represent the predicted methylated read counts of postsplicing RNA, and $s_{ij}^w( t )$ represent the predicted nonmethylated read counts of postsplicing RNA for the ${{i}^{th}}$ site and ${{j}^{th}}$ cell. We can then calculate various metrics to quantify methylation levels based on these values.

### Non-PCA-based visualization

UMAP and *t*-SNE, as nonlinear visualization tools, provide a more nuanced view of the data’s structure. By combining the current postsplicing RNA metrics of methylation levels with predictions, a visualization based on these combined metrics can be created. However, integrating predicted data can affect the embedding, making the results dependent on the accuracy of the predictions. This dependency can complicate the interpretation of changes, as any modification in predictions can alter the embedding. To mitigate the influence of predicted data and create a more robust visualization, we calculate the transition probability of the cells to generate a velocity-based visualization. This approach aims to reduce the impact of predicted data on the embedding. Two methods can be used to calculate the transition probability.

Euclidean distance method: This method calculates the transition probability based on the Euclidean distance between the differences in predictions and current metrics across cells.


(13) \begin{eqnarray*}
{P}_{ij} &=& {\mathrm{exp}}-\left(\frac{-\sum \nolimits_k {\left({m}_{ki}^s(t) - m_{kj}^{s}\right)}^{2}}{2{\sigma}^{2}} \right)\\&&\Bigg/\sum \limits_j {\mathrm{exp}}\left(\frac{-\sum \nolimits_k {\left({m}_{ki}^s(t) - m_{kj}^{s}\right)}^{2}}{2{\sigma}^{2}} \right)
\end{eqnarray*}

Here, $m_{ki}^s( t )$ is the predicted metric of the postsplicing RNA methylation level for the ${{k}^{th}}$ site of the ${{i}^{th}}$ cell (see Supplementary Note: *Theoretical framework and computational details—Metrics for RNA methylation level quantification* for metric details), $m_{kj}^s$ is the current metric for the ${{k}^{th}}$ site of the ${{j}^{th}}$ cell, and $\sigma$ is a hyper-parameter. ${{P}_{ij}}$ is the transition probability, based on gaussian kernel of the Euclidean distance.

Pearson correlation coefficient method: This method calculates the transition probability based on the Pearson correlation between the differences in predictions and current metrics, as well as the differences in current metrics across cells.


(14) \begin{eqnarray*}
{{P}_{ij}} = {\mathrm{exp}}\left( {\frac{{{\mathrm{corr}}\left( {{{r}_{ij}},{{d}_i}} \right)}}{\sigma }} \right)/\mathop \sum \limits_j {\mathrm{exp}}\left( {\frac{{{\mathrm{corr}}\left( {{{r}_{ij}},{{d}_i}} \right)}}{\sigma }} \right)
\end{eqnarray*}

(15) \begin{eqnarray*}
{{r}_{k,ij}} = {\mathrm{sgn}}\left( {m_{kj}^s - m_{ki}^s} \right)\sqrt {\left| {m_{kj}^s - m_{ki}^s} \right|}
\end{eqnarray*}

(16) \begin{eqnarray*}
{{d}_{k,i}} = {\mathrm{sgn}}\left( {m_{ki}^s(t) - m_{ki}^s} \right)\sqrt {\left| {m_{ki}^s(t) - m_{ki}^s} \right|} \
\end{eqnarray*}

Here, ${\mathrm{sgn}}()$ denotes the sign function, ${{r}_{ij}}$ is the vector measuring the difference between the ${{i}^{th}}$ cell and ${{j}^{th}}$ cell, and ${{d}_i}$ is the vector measuring the difference between the prediction and the current metric of the ${{i}^{th}}$ cell. ${{P}_{ij}}$ is the transition probability, represented as an exponential kernel of the Pearson correlation coefficient.

Assuming we have the embedding value ${{x}_i}$ for the ${{i}^{th}}$ cell, the cell’s velocity can be determined as follows:


(17) \begin{eqnarray*}
\Delta {{x}_i} = \mathop \sum \limits_j \left( {{{P}_{ij}} - \frac{1}{n}} \right)\frac{{\left( {{{x}_j} - {{x}_i}} \right)}}{{\|{{x}_j} - {{x}_i}\|}}
\end{eqnarray*}

Subtracting $\frac{1}{n}$ helps avoid a uniform distribution. Once $\Delta {{x}_i}$ is calculated, we can visualize the velocity of the methylation level, and the learned velocity can further be applied to simulate the start and end points (see *Supplementary Note: Theoretical framework and computational details—Diffusion start and end point*).

### Visualization for large dataset

For large datasets with numerous cells, visualizing the velocity for each cell individually may be impractical. Instead, we can apply a Gaussian kernel to summarize the velocity at selected grid points.


(18) \begin{eqnarray*}
\Delta {{x}_{\textit{grid}}} = \mathop \sum \limits_i {\mathrm{exp}}\left( {\frac{{ - \|{{x}_{\textit{grid}}} - {{x}_i}{{\|}^2}}}{{2{{\sigma }^2}}}} \right)\Delta {{x}_i}
\end{eqnarray*}

Here, ${{x}_{\textit{grid}}}$ represents the embedding of the grid point. This approach effectively condenses and represents the velocity data, making it feasible to visualize and interpret the dynamics of large cell populations.

## Results

### VeloRM disentangles the presplicing and postsplicing epitranscriptomes

**Figure 1.**
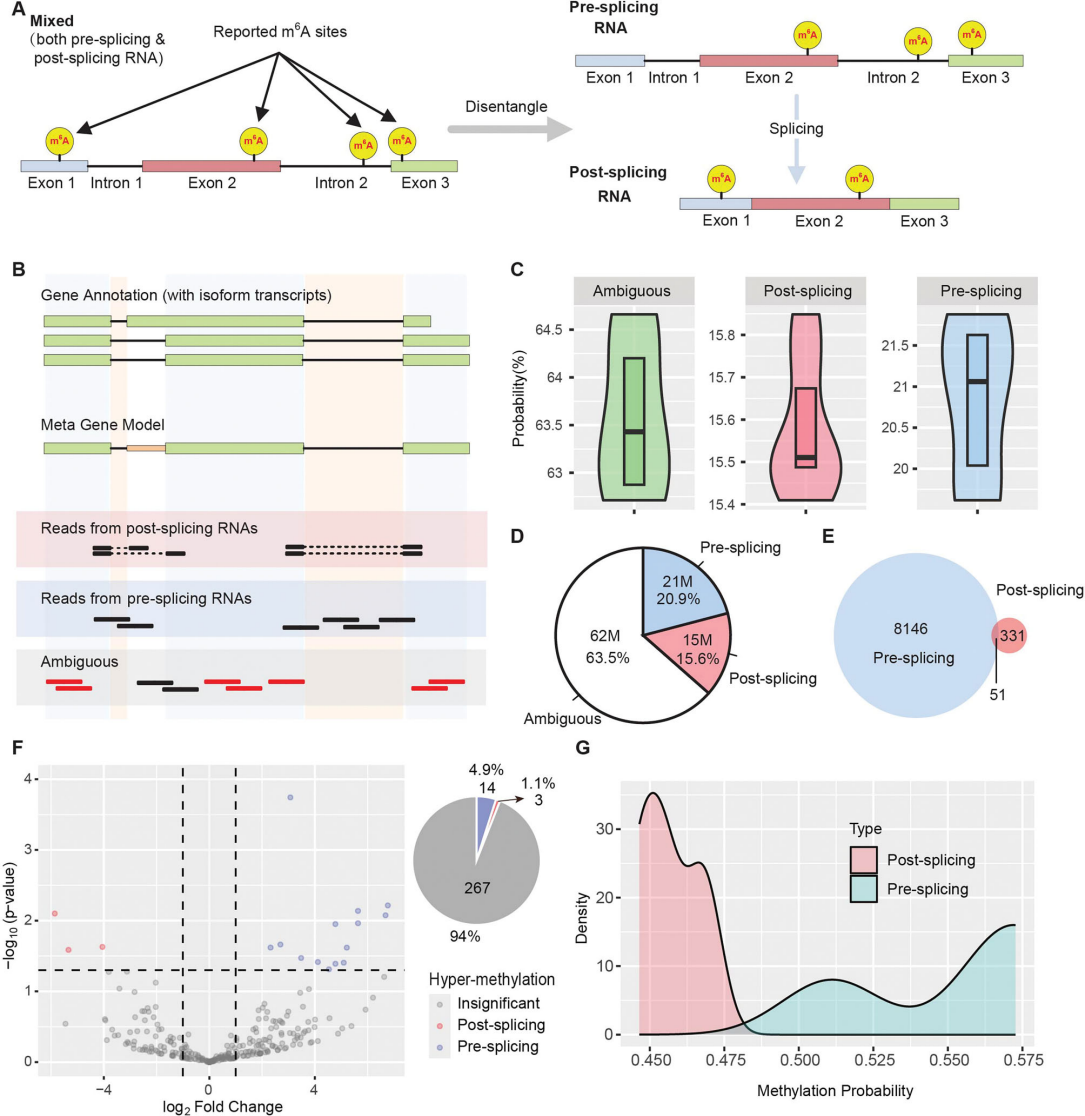
Disentangling the presplicing and postsplicing epitranscriptomes. (**A**) Schematic of existing approaches reporting a mixture of presplicing and postsplicing RNA modification sites. (**B**) Accurate separation of sequencing reads originated from presplicing and postsplicing RNAs is the key to resolving signals from the presplicing and postsplicing epitranscriptomes. (**C**) Proportions of presplicing, postsplicing, and ambiguous RNA reads vary across individual cells in the DART-seq dataset analyzed. (**D**) Aggregated read assignments across all cells. (**E**) Using DESeq2, 8146 m^6^A sites were detected exclusively in presplicing RNAs, whereas 331 sites were found only in postsplicing RNAs. (**F**) Volcano plot showing differential m^6^A methylation between presplicing and postsplicing RNAs. Fourteen sites are significantly hypermethylated in presplicing RNAs, whereas three sites are hypermethylated in postsplicing RNAs. (**G**) Density distribution of methylation proportions for presplicing and postsplicing RNAs in DART-seq (aggregated across three replicates), indicating overall higher methylation levels in presplicing RNAs. Total number of sites per group: 284.

Reliable extraction of presplicing and postsplicing epitranscriptomes—that is, RNA modifications on RNA molecules before and after splicing—is essential for studying the dynamics of RNA modifications throughout the RNA life cycle. However, to the best of our knowledge, existing epitranscriptome analysis frameworks report only a composite set of modification sites overlaying the signals from all RNAs, without distinguishing their specific RNA life stage (Fig. 1A).

Disentangling presplicing and postsplicing epitranscriptomes requires a clear separation of sequencing reads derived from the presplicing and postsplicing RNAs, respectively. The presence of multiple transcript isoforms per gene poses a major challenge, particularly when reads overlap with intronic and exonic regions belonging to different isoforms [86]. Previous efforts addressed a similar issue in RNA velocity analysis by separating the spliced and unspliced reads based on gene annotations [59], which proved highly effective for contrastive quantification of the spliced and unspliced RNA abundance. However, the study of RNA modifications demands higher precision in read classification, as the modification sites themselves need to be localized *de novo*. To this end, we proposed an improved read assignment strategy. As illustrated in Fig. 1B, our method moves many previously misassigned reads into more appropriate categories, i.e. reads aligning previously exclusively to exonic regions may originate from either presplicing or postsplicing RNAs and should therefore be labeled as ambiguous, rather than being assigned to postsplicing RNAs as by previous approach [60]. This enhanced strategy enables correct separation of epitranscriptome signals from presplicing and postsplicing RNAs and is thus critical for the following analysis (also see the ‘Annotation of reads derived from presplicing and postsplicing RNAs’ section). We adopt a conservative classification strategy in which reads that cannot be unambiguously assigned are labeled as ambiguous and excluded. This reduces data retention but prioritizes specificity and minimizes misclassification, which is critical for accurate identification of RNA modification sites (Supplementary Fig. 11 shows that probabilistic reassignment of ambiguous reads does not substantially alter the inferred trajectories, indicating robustness of the analysis). We further performed a parameter sweep in the DART-seq dataset (GSE125780), relaxing the strict pure-intronic/pure-exonic rule to allow read-overlap thresholds of 0.01–0.2. Relaxed thresholds did not improve recovery relative to the strict baseline and introduced very few new sites, with the estimated FDR proxy remaining low across thresholds (Supplementary Table 5). These results support retaining the strict ambiguous-read filter as a conservative read-assignment strategy.

Standard RNA-seq and many DRS protocols often prioritize mature transcripts through poly(A)+ selection, which can limit the recovery of presplicing RNA intermediates [87, 88]. While some DRS applications can be adapted for poly(A)-independent capture, DART-seq demonstrates an enhanced capacity to profile full-length precursor transcripts (pre-messenger RNA) alongside mature RNAs [69], offering a more comprehensive view of the transcriptome’s modification landscape across different processing stages. To evaluate this, we first analyzed a m^6^A dataset generated from HEK293T cells using DART-seq (GSE125780) [69]. For each of the three replicates per condition (six samples in total), varying proportions of presplicing and postsplicing RNA reads were extracted. On average, ∼62%–65% of reads were ambiguous in origin, 15.4%–16% were clearly derived from postsplicing RNAs, and 19%–22% could be confidently assigned to presplicing RNAs (Fig. 1C). When aggregating data across all cells, about 15.6% of reads (15 million) were assigned to postsplicing RNAs, and 20.9% (21 million) to presplicing RNAs, while the remaining 63.5% (62 million) were ambiguous, lacking clear association with either RNA type (Fig. 1D). The large proportion of ambiguous reads directly reflected the challenges of distinguishing the presplicing and postsplicing origin of sequencing reads and is a necessary cost for rigorous separation of RNA modification signals.

We initially extracted 38 899 candidate m^6^A sites and applied the DESeq2 method [78] separately to the presplicing and postsplicing RNA datasets (see the ‘Extraction of m^6^A signals from DART-seq, scDART-seq, and DRS’ section). Among these, 8146 sites were detected exclusively in presplicing RNAs, whereas 331 sites were found only in postsplicing RNAs, providing our first glimpse into the distinct landscapes of the presplicing and postsplicing epitranscriptomes (Fig. 1E).

### Direct comparison of the presplicing and postsplicing epitranscriptomes

Understanding the dynamics of RNA modifications throughout the RNA life cycle requires a direct comparison between the presplicing and postsplicing epitranscriptomes. To ensure a fair and meaningful contrast, we restricted our analysis to transcript regions supported by at least one read in both presplicing and postsplicing RNAs. This stringent filtering yielded 284 candidate m^6^A sites (see the ‘Comparison of the presplicing and postsplicing m^6^A epitranscriptomes’ section). Because presplicing and postsplicing reads differ in structure and length, this criterion inherently enriches for exonic sites located near splice junctions—regions where both RNA types share overlapping coverage (Supplementary Fig. 1A–C). We then employed DESeq2-based generalized linear model (GLM) [78] to compare m^6^A levels between presplicing and postsplicing RNAs across these junction-proximal sites. As shown in Fig. 1F, consistent with our previous findings, a greater number of m^6^A sites exhibit hypermethylation in presplicing RNAs relative to postsplicing RNAs. Similarly, analysis using a paired binomial test yielded comparable results (see Supplementary Fig. 9A). Furthermore, the overall methylation proportion was consistently higher in presplicing RNAs than in postsplicing RNAs at these junction-proximal regions (Fig. 1G).

**Figure 2.**
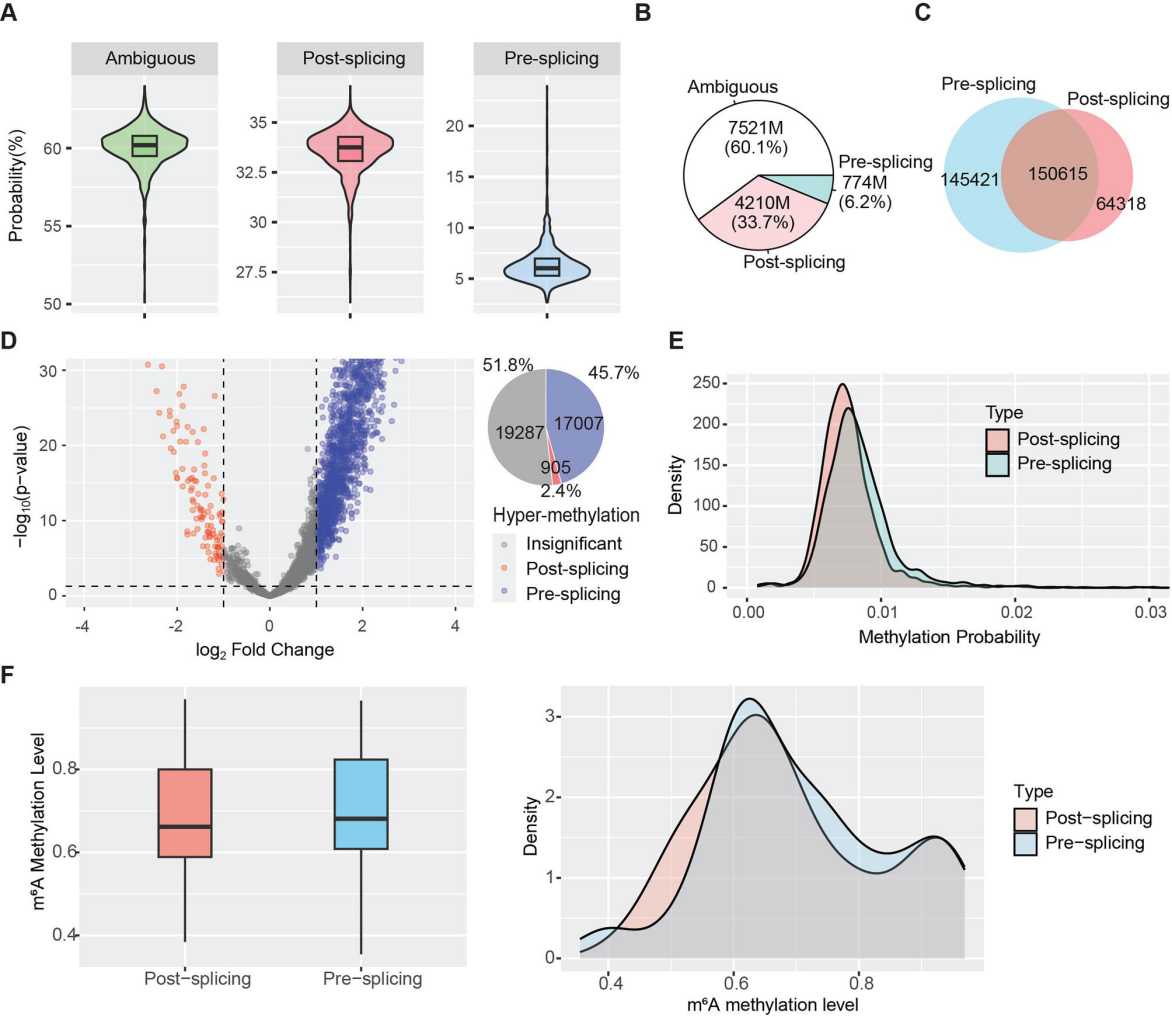
Comparative analysis of m^6^A methylation between presplicing and postsplicing RNAs. (**A–E**) scDART-seq. (**A**) Variation in the proportions of presplicing and postsplicing RNA reads across individual cells in the scDART-seq dataset. (**B**) Aggregated read composition across all cells. (**C**) Number of m^6^A sites detected in presplicing, postsplicing, or both RNA types. (**D**) Volcano plot of differential methylation analysis between presplicing and postsplicing RNAs, identifying 17 007 sites significantly hypermethylated in presplicing RNAs and 905 sites in postsplicing RNAs. (**E**) Distribution of methylation proportions in scDART-seq, showing higher levels in presplicing RNAs (1209 cells, 37 199 sites). (**F**) Direct RNA nanopore sequencing. Methylation levels at shared m^6^A sites (235) in direct RNA nanopore sequencing showed higher methylation in presplicing RNAs. Boxplot and density represent site-level values without replicates.

We next validated these observations using an independent HEK293T dataset generated from scDART-seq [44]. For each single cell, varying proportions of presplicing and postsplicing RNA reads were extracted. On average, ∼58%–62% of reads were ambiguous in origin, 33%–34% were clearly derived from postsplicing RNAs, and 5%–7% could be confidently assigned to presplicing RNAs (Fig. 2A). When aggregating data across all cells, about 33.7% of reads (4.21 billion) were assigned to postsplicing RNAs, and 6.2% (774 million) to presplicing RNAs, while the remaining 60.1% (7.52 billion) were ambiguous, lacking clear association with either RNA type (Fig. 2B).

From reads with clear classification, we identified 150 615 m^6^A sites detected in both presplicing and postsplicing RNAs, along with 145 421 presplicing-specific and 64 318 postsplicing-specific sites using the SigRM method [79] (Fig. 2C; see the ‘Extraction of m^6^A signals from DART-seq, scDART-seq, and DRS’ section). For rigorous comparison, we further focused on 37 199 high-confidence candidate m^6^A sites with sufficient read coverage (median ≥ 10) in both RNA types across all single cells (see the ‘Comparison of the presplicing and postsplicing m^6^A epitranscriptomes’ section). Applying the same DESeq2-based GLM [78] to this dataset revealed a consistent pattern: more m^6^A sites were hypermethylated in presplicing RNAs relative to postsplicing RNAs at the junction-proximal regions (Fig. 2D; details in Supplementary Table 1; paired binomial test: Supplementary Fig. 9C), with presplicing RNAs also showing higher overall methylation levels (Fig. 2E). Importantly, when examining the relationship between methylation level and distance to the nearest splice junction, we observed no systematic effect (Supplementary Fig. 2).

In combination, these analyses across DART-seq and scDART-seq consistently demonstrate that presplicing RNAs are more heavily decorated with m^6^A modifications across the junction-proximal sites, highlighting dynamic remodeling of the m^6^A landscape during RNA maturation.

We further validated this observation using an independent DART-seq dataset, which showed a consistent trend (Supplementary Fig. 3). To evaluate whether this presplicing enrichment also extends beyond junction-proximal regions, we analyzed long-read DRS data. Globally, DRS identified substantially more m^6^A sites in postsplicing RNAs (13 018 sites) than in presplicing RNAs (383 sites), likely reflecting poly(A)+ selection favoring mature transcripts. Consistent with this bias, 75.1% of classifiable DRS reads were assigned to postsplicing RNAs, and the total exon-aligned read length was 3.76-fold higher for postsplicing than presplicing reads. Further analyses using non-poly(A)-selected protocols or full-length transcript capture would be required to assess whether presplicing RNAs are globally more methylated. To mitigate this detection bias, we focused on the 235 sites detected in both presplicing and postsplicing RNAs, which primarily correspond to regions near splice junctions. At these shared sites, methylation levels were consistently higher in presplicing RNAs than in postsplicing RNAs (two-sided *t*-test, *P* = 3.68 × 10^−4^; Fig. 2F). Consistent with this trend, analysis of the same 37 199 high-confidence scDART-seq m^6^A sites in MeRIP-seq data showed a similar pattern, with higher signal observed in presplicing RNAs than in postsplicing RNAs (Supplementary Fig. 3; see Supplementary Note for details). However, given the peak-level resolution of MeRIP-seq, this analysis serves only as complementary support rather than independent validation.

Together, these results indicate that presplicing RNAs are preferentially methylated near splice junctions, while global transcript-wide trends may be influenced by read coverage and detection biases.

### VeloRM uncovers the single-cell velocity of m^6^A during cell cycle

**Figure 3.**
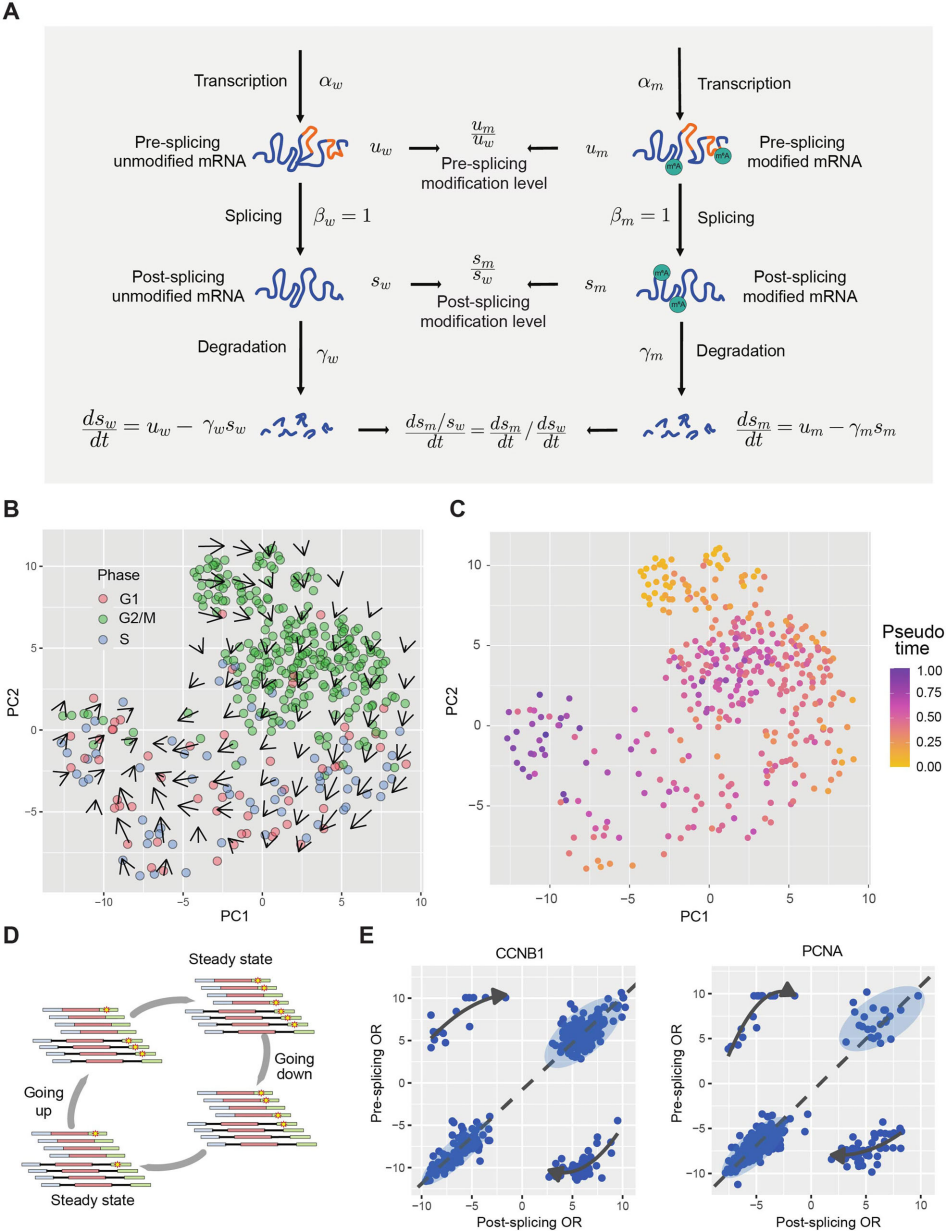
Transient single-cell dynamics of m^6^A methylation during cell cycle. (**A**) Conceptual model of epitranscriptome dynamics incorporating transcription (${{\alpha }_m},{{\alpha }_w})$, splicing (${{\beta }_m},{{\beta }_w})$, and degradation rates (${{\gamma }_m},{{\gamma }_w}$). The model tracks methylated and unmethylated transcripts in both presplicing and postsplicing forms, capturing the transient behavior of m^6^A methylation. (**B**) Phase portrait of global m^6^A dynamics across the cell cycle (aggregated 142 sites, Gaussian smoothing). (**C**) Pseudo-time estimation of cell cycle progression using a transition matrix (0 = start, 1 = end). (**D**) Two patterns of steady-state methylation transitions observed at single sites. (**E**) Representative m^6^A sites (chr5:69 174 992, +, *CCNB1*; chr20:5 115 239, −, *PCNA*) showing dynamic transitions in methylation levels.

To characterize the transient dynamics of RNA methylation, we developed a differential equation model describing the time-dependent relationship between RNA methylation levels on presplicing and postsplicing RNAs. We first separated methylated and unmethylated signals, then modeled the first-order time derivative of postsplicing RNA abundance (i.e. RNA velocity) as the net balance between its production from presplicing RNA and its degradation, following the framework of [59]. By comparing the transient behaviors of methylated and unmethylated RNAs, the kinetics of m^6^A methylation levels along pseudo time can be inferred (Fig. 3A; see the ‘Theory and computational methods’ section; *Supplementary Note: Theoretical framework and computational details*).

To demonstrate the effectiveness of the proposed framework in predicting the transient dynamics of RNA methylation in the single cells, we analyzed the scDART-seq m^6^A datasets [44] and applied it to the 142 m^6^A sites on cell cycle related genes as previously described [79], and the details of the selection process are provided in the ‘Characterization of transient m^6^A dynamics during cell cycle progression’ section. In order to visualize the overall dynamics of m^6^A modification during cell cycle progression, we generated phase portraits based on the aggregated methylation signals of the 142 selected sites using Gaussian smoothing on a regular grid. The resulting trajectory revealed a cyclic pattern highly consistent with the known cell states, which were annotated by the expression of cell cycle marker genes [79], with cells transitioning from G2/M to G1/S and looping back to G2/M (Fig. 3B). The separation between G1 and S phases appeared less distinct, possibly due to the limited specificity of known marker genes for distinguishing these phases [79]. Meanwhile, the transient epitranscriptomic dynamics at single-cell resolution, derived from the same aggregated methylation signals without smoothing is shown in Supplementary Fig. 4A. The corresponding scVelo [61] RNA velocity field is provided in Supplementary Fig. 4B. While the fine-scale patterns differ—likely due to the distinct information layers captured by the two approaches (methylation versus RNA abundance)—the overall trajectories are highly consistent. These complementary layers of information together provide a more holistic view of the underlying cellular dynamics.

To evaluate the robustness of our site selection, we performed an ablation test comparing the 142 selected sites to all sites, scVelo, and randomly selected sites. The phase portrait for all sites is shown in Supplementary Fig. 6A, with the corresponding *t*-SNE visualization in Supplementary Fig. 6B. In the UMAP-based evaluation, the cosine similarity of the trajectory inferred from the selected 142 sites was 0.763, compared with 0.706 for all sites, 0.624 for scVelo, and a mean of 0.701 (standard deviation 0.044) for random site selections. The selected sites showed a modest improvement over random site selections in the UMAP embedding (empirical *p* = 0.0792), with a stronger enrichment observed in the *t*-SNE embedding (empirical *p* = 0.0099). The ablation test results based on cosine similarity are summarized in Supplementary Fig. 7A and B.

Furthermore, we constructed a transition matrix and visualized the estimated states. As shown in Fig. 3C, individual cells were assigned a pseudo-time value ranging from 0 to 1, with lower values representing likely starting points and higher values indicating terminal states, which is again consistent with the expected cyclic nature of cell cycle progression. While the primary trajectory was inferred using UMAP [81] embeddings, a *t*-SNE [82] visualization is also provided in Supplementary Fig. 4C–F.

At the single-site level, two distinct patterns of methylation dynamics were observed based on aggregated methylation signals, as shown in Fig. 3D. In the first pattern, methylation levels increased from a low to a high steady state. In this case, the methylation level of presplicing RNA began to rise, and continued to supersede postsplicing RNA in the process. In the second pattern, methylation levels decreased from a high to a low steady state. Here, the methylation level of presplicing RNA declined first, and this trend then passed to postsplicing RNA. Both patterns reflect the joint effects of splicing-driven initiatives and the degradation processes. We further show in Fig. 3E two representative m^6^A sites (chr5:69 174 992, +, *CCNB1*) and (chr20:5 115 239, –, *PCNA*) to illustrate these steady-state transitions, highlighting the dynamic behavior of m^6^A at the single-site level within a periodic process. Notably, as illustrated by the representative *CCNB1* site in Supplementary Fig. 12, key moments of RNA modification state transitions are clearly captured, highlighting the temporal resolution of our approach.

### VeloRM tested on single-cell A-to-I RNA editing dataset

#### Chromaffin cell differentiation dataset

**Figure 4.**
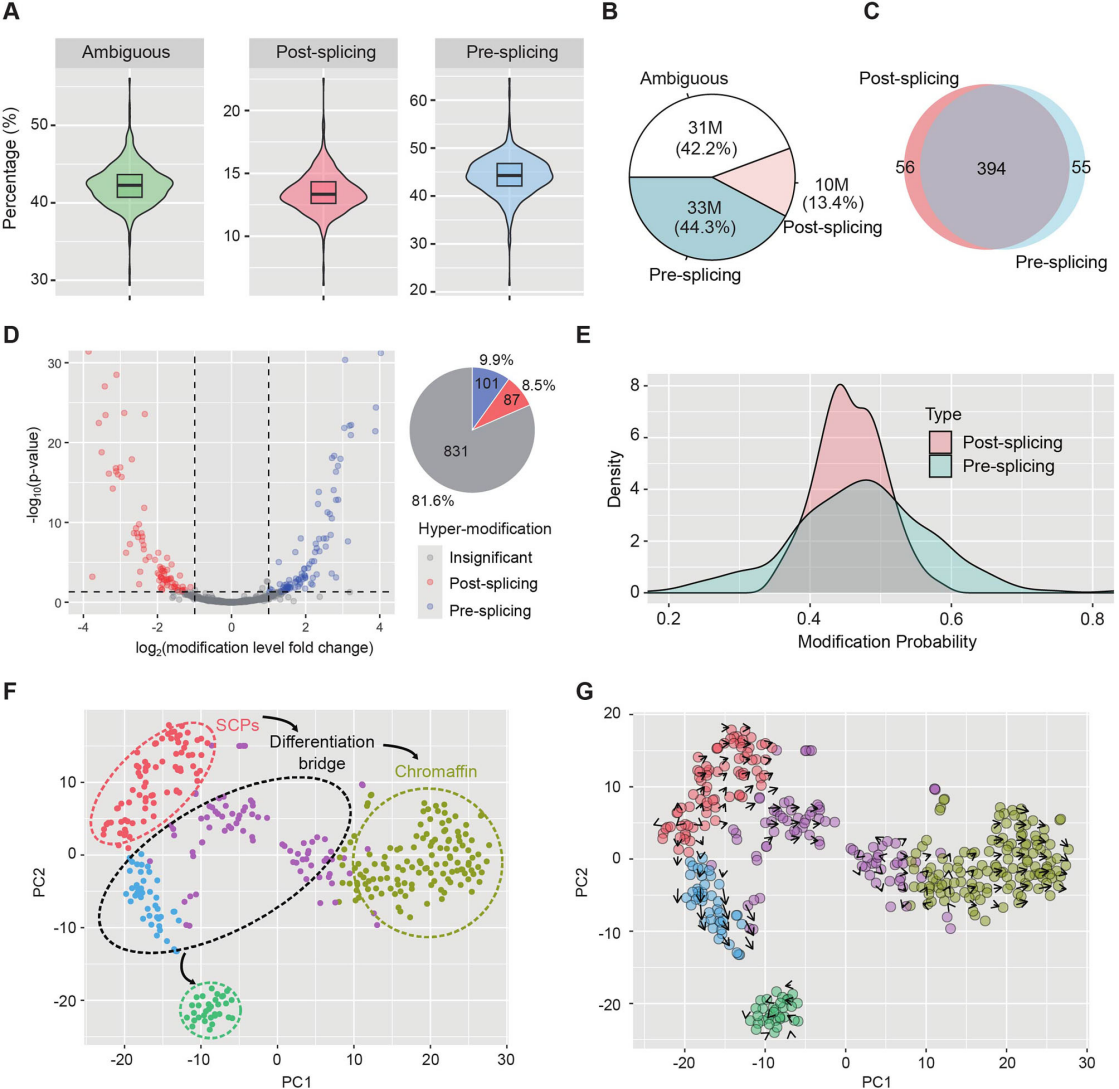
Comparative analysis and dynamic regulation of A-to-I RNA editing in the single-cell chromaffin cell differentiation dataset. (**A**) Variation in proportions of presplicing, postsplicing, and ambiguous reads across single cells. (**C**) Number of A-to-I editing sites detected in presplicing, postsplicing, or both RNA types. (**D**) Differential RNA editing analysis showing no strong bias between presplicing and postsplicing RNAs. (**E**) Distribution of A-to-I editing proportions at the single-cell level, indicating similar overall levels between RNA types (384 cells, 1019 sites). (**F**) Cell differentiation trajectory visualized using gene-expression embeddings from the original study, rather than embeddings derived from RNA editing profiles, showing progression from Schwann cell precursors (SCPs) to chromaffin cells. (**G**) RNA editing-based velocity field inferred from 36 selected sites and visualized on the same gene-expression embedding, capturing a coherent differentiation trajectory consistent with gene expression-based inference.

We also applied VeloRM to a single-cell A-to-I dataset [83] generated from SMART-seq2 platform, which was originally used to profile the differentiation trajectory of adrenergic chromaffin cells. After separating reads into presplicing and postsplicing RNAs, ∼41%–44% of reads were ambiguous in origin across individual single cells, 13%–14% were clearly derived from postsplicing RNAs, and 42%–47% could be confidently assigned to presplicing RNAs (Fig. 4A). When aggregating data across all cells, 13.4% of reads (10 million) were clearly assigned to postsplicing RNAs, and 44.3% (33 million) to presplicing RNAs, while the remaining 42.2% (31 million) were ambiguous, lacking confident association with either RNA type (Fig. 4B). The large proportion of ambiguous reads, similar to those in the scDART-seq data, underscores again the challenge of distinguishing presplicing RNAs.

We then performed a direct comparison between presplicing and postsplicing A-to-I editomes to investigate their regulatory differences. From the scRNA-seq dataset [83], we identified 1019 candidate A-to-I RNA editing sites that are observable on both presplicing and postsplicing RNAs (see the ‘A-to-I RNA editing site detection from single cell RNA-seq data’ section). Among the 1019 sites, 394 were significant on both the presplicing and postsplicing RNAs, while 55 and 56 sites were uniquely detected on presplicing and postsplicing RNAs, respectively. (The remaining A-to-I sites are insignificant on either postsplicing or presplicing RNAs.) Overall, the number of A-to-I sites are similar between presplicing and postsplicing RNAs (Fig. 4C).

Differential analysis was then conducted with DESeq2 [78] to compare the editing signals between postsplicing RNA and presplicing RNA. As illustrated in Fig. 4D and paired binomial test in Supplementary Fig. 10A, the differentially edited sites show no strong bias toward either RNA type; the number of significant sites in postsplicing RNA and presplicing RNA was comparable. We further examined the distribution of editing proportions at the single-cell level and found that the overall editing levels were similar between the two RNA types (Fig. 4E). The observed differences in variance could be attributed to differences in read depth. Notably, A-to-I editing levels in presplicing RNAs were not affected by the distance to the nearest splice junction (Supplementary Fig. 2). In postsplicing RNAs, we observed a slight increase in editing levels with greater distance from the splice junction; however, this trend may reflect a technical bias, as regions farther from junctions are covered by fewer reads.

We further leveraged RNA editing site data to investigate transient dynamics in the single-cell A-to-I epitranscriptome and to reconstruct the cell differentiation trajectory. Cell phase labels corresponding to the transition from SCPs to chromaffin cells were obtained from a previously published study [83]. A total of 36 A-to-I editing sites associated with cell cycle markers were selected for the analysis (see the ‘Characterization of transient A-to-I RNA editing dynamics during cell differentiation progression’ section). As shown in Fig. 4F, the differentiation process from SCPs to chromaffin cells was visualized using previously published gene-expression embeddings from [59], rather than embeddings derived from RNA editing profiles. Building on this expression-based embedding, we aimed to reconstruct the same process using the 36 selected A-to-I editing sites. To capture the general dynamic patterns across the cell population, we visualized single-cell RNA editing velocities using locally averaged vector fields (Fig. 4G), while single-cell-level velocities were presented in Supplementary Fig. 5A, and the corresponding scVelo [61] RNA velocity is provided in Supplementary Fig. 5B. The resulting epitranscriptome trajectory revealed a clear progression from SCPs to chromaffin cells, as well as the emergence of a distinct subpopulation, aligning well with the temporal ordering observed in Fig. 4F. Notably, an ablation test for site selection is provided in Supplementary Figs 6C and 7C. The Spearman correlation was 0.433 for the 36 selected sites, compared with 0.351 for all sites, 0.271 for scVelo, and a mean of 0.384 with a standard deviation of 0.055 for random site selections. The selected sites showed a higher correlation than the random mean, with a permutation-based empirical *P*-value of 0.218, consistent with a modest trend.

### Multicell-type broad RNA spectrum dataset

**Figure 5.**
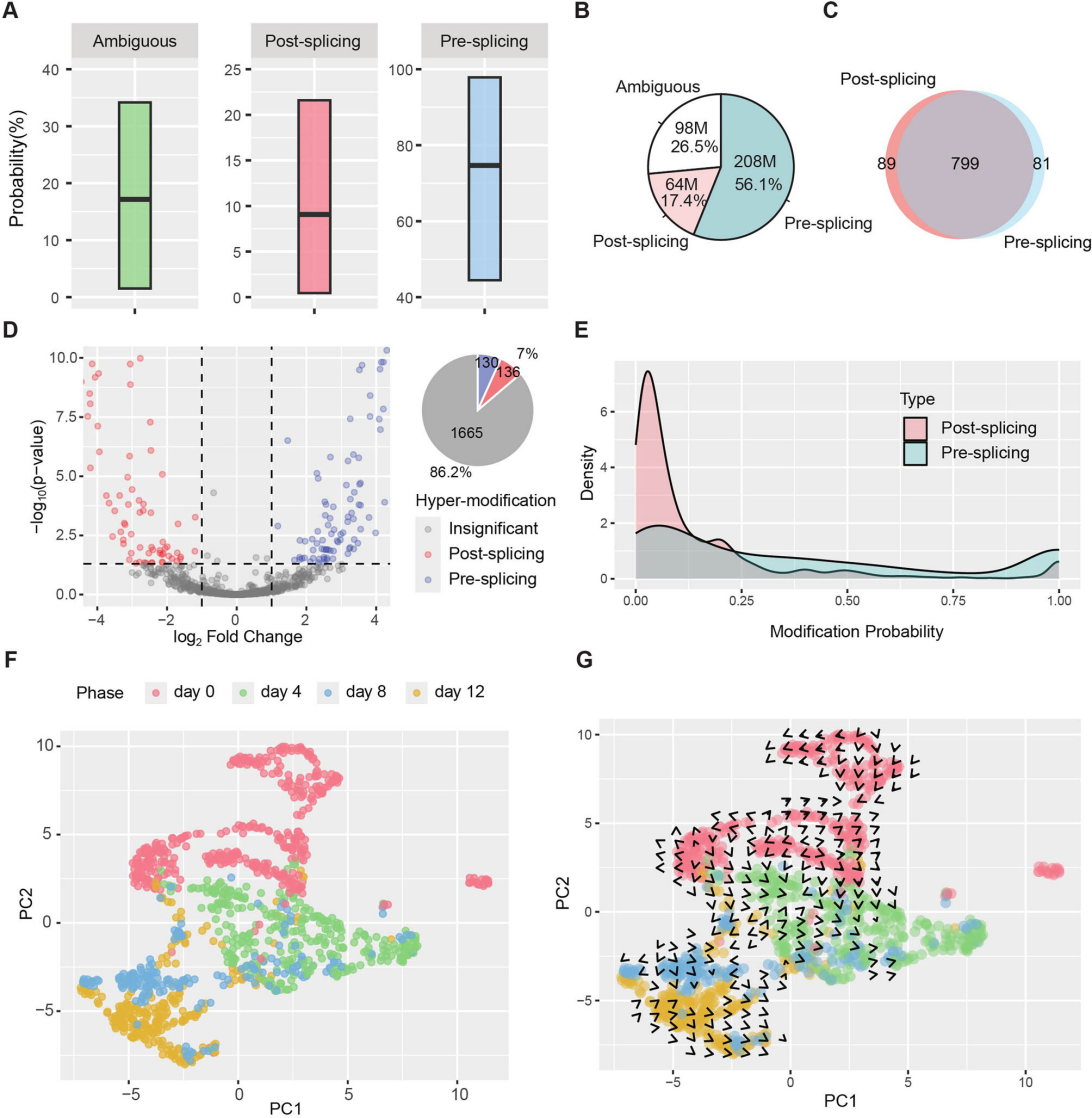
Comparative analysis and dynamic regulation of RNA processing dynamics in the mESC EB differentiation dataset. (**A**) Variation in proportions of presplicing, postsplicing, and ambiguous reads across single cells. (**B**) Aggregated read composition across all cells. (**C**) Number of A-to-I editing sites detected in presplicing, postsplicing, or both RNA types. (**D**) Differential analysis showing no strong global bias between presplicing and postsplicing RNAs. (**E**) Distribution of A-to-I editing proportions at the single-cell level, indicating broadly similar levels between RNA types (1679 cells, 1931 sites). (**F**) Differentiation trajectory visualized using gene-expression embeddings from the original study, rather than embeddings derived from RNA editing profiles, showing progression from pluripotent mESCs (day 0) through EB stages (day 4, 8, 12). (**G**) RNA editing-based velocity field inferred from 27 selected sites and visualized on the same gene-expression embedding, capturing a coherent differentiation trajectory consistent with gene expression-based inference.

We also applied VeloRM to a broad RNA spectrum single-cell dataset generated from mouse embryonic stem cells (mESC), originally used to characterize non coding RNA patterns across differentiation [85]. After separating reads into presplicing and postsplicing RNAs, we observed substantial variability across single cells, with 2%–34% of reads being ambiguous, 1%–23% clearly originating from postsplicing RNAs, and 43%–96% confidently assigned to presplicing RNAs (Fig. 5A). When aggregated across all cells, 17.4% of reads (64 million) were assigned to postsplicing RNAs, 56.1% (208 million) to presplicing RNAs, and 26.5% (98 million) remained ambiguous (Fig. 5B).

We next compared presplicing and postsplicing A-to-I editomes. From this dataset [85], we identified 1931 A-to-I candidate sites detectable in both RNA types (see the ‘A-to-I RNA editing site detection from single cell RNA-seq data’ section). Among them, 799 sites were significant in both presplicing and postsplicing RNAs, whereas 89 and 81 sites were uniquely significant in presplicing and postsplicing RNAs, respectively; the remaining sites were insignificant in either RNA type. Overall, the number of A-to-I sites was similar between the two RNA types (Fig. 5C), consistent with the observations in the chromaffin differentiation dataset.

Differential analysis with DESeq2 [78] revealed no strong directional bias: significant sites were comparable between presplicing and postsplicing RNAs (Fig. 5D and paired binomial test in Supplementary Fig. 10B). At the single-cell level, the distribution of editing proportions was also similar between the two RNA types (Fig. 5E), with differences in variance likely explained by read-depth variation.

We then leveraged the editing-site data to examine transient dynamics along the mESC embryoid-body (EB) differentiation time course and to reconstruct the developmental trajectory. Differentiation stage labels corresponding to day 0 mESCs and day 4, day 8, and day 12 EBs were taken from the original study [85]. A total of 27 cell-cycle-associated A-to-I editing sites were selected for downstream modeling (see the ‘Characterization of transient A-to-I RNA editing dynamics during cell differentiation progression’ section). As shown in Fig. 5F, the differentiation progression across the four stages was visualized using previously published gene-expression embeddings from [85], rather than embeddings derived from RNA editing profiles. Using the selected editing sites on this expression-based embedding, we reconstructed the same progression by visualizing locally averaged RNA editing velocity fields (Fig. 5G), with single-cell-level velocities shown in Supplementary Fig. 5C and the corresponding scVelo [61] RNA velocity provided in Supplementary Fig. 5D. The resulting epitranscriptomic trajectory recapitulated the expected ordering along the differentiation series, demonstrating a clear transition from undifferentiated mESCs through successive EB stages. Notably, an ablation test for site selection is provided in Supplementary Figs 6D and 7D. The Spearman correlation was 0.598 for the 27 selected sites, compared with 0.604 for all sites, 0.293 for scVelo, and a mean of 0.534 with a standard deviation of 0.105 for random site selections. The selected sites performed comparably to all sites and above the random mean, with a permutation-based empirical *P*-value of 0.317, consistent with a modest trend.

## Discussion

RNA modification serves as a critical regulator of gene expression, influencing diverse biological processes and disease mechanisms. With advances in single-cell technologies, transcriptome-wide RNA methylation can now be profiled across thousands of cells, enabling unprecedented insights into its functional roles in dynamic systems and heterogeneous populations. A major conceptual advancement in single-cell analysis is RNA velocity, which infers future transcriptional states by comparing presplicing and postsplicing transcripts, offering powerful tools for trajectory reconstruction and cellular dynamics analysis. However, methods tailored for RNA modification kinetics at single-cell resolution are currently scarce.

To address this gap, we develop VeloRM, a computational framework for modeling the dynamics of single-cell RNA modifications such as m^6^A and A-to-I at single-nucleotide resolution. VeloRM extends the concept of RNA velocity to the epitranscriptome by quantifying the gain and loss of modifications over pseudotime. This enables direct investigation of modification kinetics during complex biological processes, offering new perspectives into epitranscriptome regulation. Applied to single-cell epitranscriptomic data, VeloRM captures modification-state transitions across developmental trajectories, identifies transient regulatory states. By integrating modification timing with cellular heterogeneity, VeloRM provides a novel lens for dissecting epitranscriptomic regulation in complex systems.

We applied VeloRM to three distinct single-cell epitranscriptomic datasets to demonstrate its analytical capabilities: one m⁶A dataset generated using scDART-seq, and two A-to-I RNA editing datasets derived from scRNA-seq—one from the chromaffin cell differentiation system and the other from the mESC EB differentiation system. In the scDART-seq m^6^A dataset, VeloRM uncovered diverse temporal modification programs. Notably, N^6^-methyladenosine (m^6^A) is more heavily decorated on presplicing RNAs than postsplicing RNAs near the splicing junctions. The difference in temporal RNA methylation patterns (before and after splicing) enabled the reconstruction of cellular trajectories and provided insights into the regulatory roles of m^6^A dynamics in a complex process. We found that m^6^A methylation was generally negatively correlated with postsplicing RNA abundance for a subset of 3086 correlated sites out of 37 199 candidates, suggesting a role in promoting RNA decay, largely driven by modifications occurring on presplicing transcripts. In contrast, a small subset of m^6^A sites showed positive correlations with postsplicing RNA levels, predominantly associated with postsplicing methylation, indicating potential functions in enhancing transcript stability or promoting RNA accumulation (see Supplementary Note: Transcriptional Impact Analysis; Supplementary Fig. 8). When applied to the A-to-I editing dataset, VeloRM similarly identified stage-specific RNA editing patterns, which enabled the inference of dynamic cellular trajectories during cell differentiation, further underscoring the utility of VeloRM in characterizing the kinetics of diverse RNA modifications at single-cell resolution.

The VeloRM model currently has several limitations. First, the model is optimized for high-depth sequencing platforms such as SMART-seq2, limiting its applicability to lower-depth platforms like 10x Genomics [49]. 10x-based protocols capture thousands of cells but have insufficient per-cell read depth for reliable site-level epitranscriptomic analysis, as accurate inference requires reads at the same nucleotide across multiple cells. Potential strategies for extending VeloRM to 10x data include pooling cells into pseudobulk profiles or performing ultra-deep sequencing. To further assess depth dependence, we performed a processed-matrix-level subsampling analysis using the 37 199 high-confidence scDART-seq m^6^A sites, which showed relatively stable trajectory recovery across moderate retained-signal fractions but reduced performance at the lowest fractions (Supplementary Fig. 14). Because this analysis was performed after site detection and matrix construction, it should be interpreted as a controlled retained-signal robustness assessment rather than an absolute raw read-depth threshold. In addition, while we identified presplicing- and postsplicing-prominent m^6^A and A-to-I RNA modification sites with distinct transcriptome distributions (MetaTX analyses [89], see Supplementary Note: Mechanism insights into the formation of presplicing- and postsplicing-prominent sites; Supplementary Fig. 1D–F; Supplementary Table 2–4), experimental validation is still required to confirm their functional relevance.

Second, a fundamental limitation of VeloRM is the assumption that splicing rates are equal for methylated and unmethylated transcripts ($\ {{\beta }_m} = {{\beta }_w} = 1$). Moreover, active demethylation is not explicitly modeled, consistent with prior observations that RNA degradation exerts a larger influence on modification levels than active demethylation [90]. Since m^6^A can influence splicing, estimated degradation rates (γ) may absorb systematic errors. To assess the sensitivity of VeloRM to this splicing-rate assumption, we performed an *in silico* robustness analysis by perturbing the modified-RNA splicing rate ${{\beta }_m}$ while keeping the nonmodified splicing rate unchanged. The trajectory-based cosine similarity and pseudotime-based Spearman correlation remained stable across the tested ${{\beta }_m}$values (Supplementary Fig. 13), suggesting that the inferred trajectories are robust to moderate ${{\beta }_m}$ misspecification. Kinetic parameters should therefore be interpreted qualitatively, and future work could model isoform- or modification-specific splicing rates. Notably, presplicing RNAs are also subject to nuclear degradation, but this is not explicitly modeled in VeloRM due to the additional modeling complexity this would entail.

Nevertheless, as VeloRM is built upon the RNA velocity framework [59], there is considerable potential for future enhancement. Integrating methodological advances from models like scVelo [61], which leverages a dynamical modeling framework to capture transient cell states and infer latent time, and DeepVelo [64], which employs graph convolutional neural networks to model complex, nonlinear transcriptional dynamics across heterogeneous cell populations, may improve its predictive accuracy and extend its applicability to broader datasets. In our analyses, trajectories inferred from scVelo RNA velocity and VeloRM epitranscriptome velocity were broadly consistent, with minor differences reflecting distinct information layers. This suggests that combining RNA velocity with epitranscriptome velocity in a multi-omics framework could provide a more comprehensive view of cellular dynamics and transient regulatory processes in future studies.

A notable limitation arises from the use of DART-seq [69] and scDART-seq [44] for m^6^A mapping. These methods rely on the binding affinity of the YTH domain for m^6^A-modified RNA; however, the YTH domain (shared across YTHDF1-3 and YTHDC1/2) also binds non-m^6^A regions with substantial frequency: studies have shown that the YTH domain exhibits only a modest fourfold binding preference for m^6^A *in vivo* [91], and CLIP-based analyses have reveal widespread nonspecific interactions [92]. This weak specificity contributes to elevated noise levels in DART-seq data and likely underlies its weaker signal enrichment compared to more selective approaches such as m^6^A-miCLIP [93].

Additionally, our A-to-I editing analysis relies on detecting A-to-G substitutions in single-cell RNA-seq data. However, A-to-G mismatches are not uniquely indicative of inosine edits and may arise from other types of RNA modifications, sequencing artifacts, or alignment errors [94], introducing ambiguity and complicating the reliable identification of genuine A-to-I events [95]. These challenges underscore the need for more refined methods to distinguish true A-to-I editing from other transcriptomic alterations [96]. In addition, unlike scDART-seq—which captures full-length presplicing RNA and postsplicing transcripts—conventional RNA-seq and DRS primarily rely on poly(A)+ selection and therefore predominantly capture postsplicing RNAs with limited representation of presplicing transcripts [87, 88].This intrinsic bias restricts the detection of precursor RNA editing events, resulting in a less complete view of the transcriptome and its modification landscape compared with DART-seq-based profiling [69].

In summary, our VeloRM framework sheds new light on the dynamics of the RNA epitranscriptome at site, transcript and cellular level. Future methodological and algorithmic innovations will enhance its value further.

## Supplementary Material

gkag645_Supplemental_Files

## Data Availability

The datasets analyzed in this study are publicly available. The raw sequencing data were downloaded from the Gene Expression Omnibus (GEO) under accession numbers GSE180954, GSE125780, GSE297551, GSE132971, GSE99933 and GSE151334. Annotated data, analysis results, and the R implementation are available at: https://doi.org/10.6084/m9.figshare.28955183. The VeloRM R package is freely accessible on GitHub: https://github.com/whz991026/VeloRM

## Code availability

All results reported in this paper and the corresponding R implementation are available at: https://doi.org/10.6084/m9.figshare.28955183.

## Lead contact

Further information and requests for resources and reagents should be directed to and will be fulfilled by the lead contact, Jia Meng (jia.meng@xjtlu.edu.cn).
