# Supplementary material for: VeloRM: disentangling pre- and post-splicing RNA modification dynamics at single-cell resolution
Source: Nucleic Acids Res. 2026 Jun 30;54(12):gkag645. doi: 10.1093/nar/gkag645 (PMC13316427; doi:10.1093/nar/gkag645)
Supplement: gkag645_Supplemental_Files [file gkag645_supplemental_files.zip › 180626055412_Supplement-v0.11.pdf]

APPENDIX

Theoretical Framework and Computational Details

This section provides additional methodological details supplementing the **Theory and Computational Methods** section in the main text.

Parameter estimation

We model site-specific degradation rates under methylated γ_i^m and non-methylated γ_i^w conditions, requiring separate parameter estimation for each genomic site. For the methylated species (as our representative case), we employ a linear regression approach based on the fundamental relationship $u_i^m \sim \gamma_i^m \cdot s_i^m$, where u_i^m is the size-normalized methylated read counts of pre-splicing RNA and s_i^m is the size-normalized methylated read counts of post-splicing mRNA for the ith site.

While this estimation is theoretically valid under steady-state conditions ($\frac{ds_{ij}^m}{dt} = 0$), the practical identification of steady-state cells presents significant challenges. To address this limitation, we introduce two biologically plausible assumptions that yield distinct sub-models for parameter estimation:

<u>i. robust quantile linear regression</u>

The robust quantile regression approach operates under the assumption that cells exhibiting extreme expression patterns, either minimal (near origin) or maximal (upper-right corner) values of $s_{ij}^m + u_{ij}^m$ most closely approximate steady-state conditions. The formal specification of this model is:

$$u_{ij}^m \sim \gamma_i^m \cdot s_{ij}^m + o_i^m,\ \ j \in J_Q \tag{1}$$

where $J_Q = \{s_{ij}^m + u_{ij}^m \geq Q(p)\} \cup \{s_{ij}^m + u_{ij}^m \leq Q(1-p)\}$ defines the quantile-restricted sample.

$$Q_X(p) = F_X^{-1}(p) \tag{2}$$

Here, $Q_X(p)$ is the quantile function, where its input p represents the percentage of the data, set to 0.95 by default. The parameter o_i^m is the optional offset parameter, which can be seen as the intercept of the linear regression. This formulation selectively utilizes the most extreme 5% of observations from each tail of the joint expression distribution for parameter estimation.

<u>ii. weighted linear regression</u>

The weighted regression framework incorporates all cellular observations while implementing a fourth-power weighting scheme to emphasize high-abundance measurements:

$$u_{ij}^m \sim \gamma_i^m \cdot s_{ij}^m + o_i,\ \ \text{with weights}\ \ w_{ij}^m = \left(u_{ij}^m\right)^4 + \left(s_{ij}^m\right)^4 \tag{3}$$

where the offset parameter is pre-estimated via:

$$\hat{o}_i = \frac{\sum u_{ij}^m}{N_o^m + 1},\ \ j \in J_L \tag{4}$$

where $J_L = \{s_{ij}^m \leq Q_X(p)\}$ defining the low-expression subset (p = 0.01 default), and N_o^m is the number of the cells satisfying $j \in J_L$.

Cell K-Nearest Neighbor (KNN) pooling

To improve the performance of estimating γ_i^m, we employ a K-nearest neighbor (KNN) pooling strategy under the assumption that transcriptionally similar cells share comparable splicing kinetics. Cellular similarity is quantified using Pearson correlation coefficients computed from normalized read count profiles, though alternative distance metrics. For each target cell, we identify its K most similar neighbors to construct a local cellular neighborhood. By pooling the read counts for each cell with its neighbors, we can increase the total read count, making the read counts more robust and reducing potential bias from small read counts.

Site K-Nearest Neighbor (KNN) pooling

Building upon the transcriptional similarity principle, we extend the K-nearest neighbor framework to genomic loci under the assumption that proximal sites with comparable splicing patterns exhibit similar degradation kinetics γ_i^m values. This assumption allows us to leverage the information from neighboring sites to improve our estimates. To implement this, we use the K-nearest neighbors (KNN) algorithm to

select the nearest K neighbors for each site based on similarity of post-splicing read counts (log(s_{ij})) in terms of Pearson correlation.

Then we employ Bayesian linear regression *[1]* with a Gaussian prior distribution. This method provides a probabilistic framework for estimating γ_i^m, allowing us to incorporate prior knowledge and uncertainty into our estimates. The formulas are as follows:

$$\gamma_i^m \sim N(\mu_i^m, (\sigma_i^m)^2) \tag{5}$$

$$\mu_i^m = \mathrm{E}_k\left[\mathrm{E}_j\left[\frac{u_{kj}^m}{s_{kj}^m}\right]\right], k \in K \tag{6}$$

$$\sigma_i^2 = \mathrm{Var}_k\left[\mathrm{E}_j\left[\frac{u_{kj}^m}{s_{kj}^m}\right]\right], k \in K \tag{7}$$

$$u_{ij}^m \sim \gamma_i^m \cdot s_{ij}^m + o_i^m \tag{8}$$

Here, K represents the set of the site and its K neighboring sites. The expectation $\mathrm{E}_j\left[\frac{u_{kj}^m}{s_{kj}^m}\right]$is calculated over the cells for each neighboring site, and the variance $\mathrm{Var}_k\left[\mathrm{E}_j\left[\frac{u_{kj}^m}{s_{kj}^m}\right]\right]$and expectation $\mathrm{E}_k\left[\mathrm{E}_j\left[\frac{u_{kj}^m}{s_{kj}^m}\right]\right]$ are computed across the neighboring sites.

This Bayesian approach leverages information from neighboring sites, increasing the robustness of read counts and reducing biases from low read counts. By pooling data from similar sites, we can obtain more accurate and reliable estimates of γ_i^m.

Metrics for RNA methylation Level Quantification

To compare methylation levels across cells, we calculate metrics that replace methylated and non-methylated reads. If only test cell data are available, we can compute Beta and M values. If control cell data are also available, we can compute the risk ratio (RR), odds ratio (OR), and off-target effect-corrected ratio (TCR). These metrics are computed separately for post-splicing mRNA and pre-mRNA to characterize methylation dynamics at different transcriptional stages. For post-splicing mRNA methylation analysis:

<u>Size Factors</u>

To compare methylation across different cells, it is crucial to normalize sequencing depth using size factors. The size factor, $\hat{e}_j$, is calculated using the geometric median method based on the DESeq approach *[2]*:

$$\hat{e}_j = \underset{i}{\mathrm{median}} \frac{S_{ij}}{(\prod_{k=1}^{N} S_{ik})^{1/(N)}} \tag{9}$$

Here, N represents the number of cells, the S_{ij} is the post-splicing mRNA read counts of the ith site and the jth cell.

Let S_{ij}^m denote the methylated post-splicing read count and S_{ij}^w denote the non-methylated post-splicing read count for the ith site and the jth test cell, with s_{ij}^m and s_{ij}^w being the size-normalized. Similarly, let C_{ik}^m and C_{ik}^w represent the methylated and non-methylated read counts of post-splicing mRNA for the ith site and the kth control cell, respectively.

<u>Beta-value</u>

The Beta-value *[3]* represents the ratio of methylation probability. The formula is:

$$Beta_{ij}^s = \frac{S_{ij}^m/\hat{e}_j}{S_{ij}^m/\hat{e}_j + S_{ij}^w/\hat{e}_j + \varepsilon} = \frac{s_{ij}^m \sum_i S_{ij}/\hat{e}_j}{s_{ij}^m \sum_i S_{ij}/\hat{e}_j + s_{ij}^w \sum_i S_{ij}/\hat{e}_j + \varepsilon} = \frac{s_{ij}^m}{s_{ij}^m + s_{ij}^w + \varepsilon} \tag{10}$$

We can use the s_{ij}^m and s_{ij}^w to calculate the Beta-value.

M-value

The M-value *[3]* is calculated as follows::

$$M_{ij}^{s} = \log_2\left(\frac{S_{ij}^{m}/\hat{e}_j + \varepsilon}{S_{ij}^{w}/\hat{e}_j + \varepsilon}\right) = \log_2\left(\frac{s_{ij}^{m}\sum_i S_{ij}/\hat{e}_j + \varepsilon}{s_{ij}^{w}\sum_i S_{ij}/\hat{e}_j + \varepsilon}\right) = \log_2\left(\frac{s_{ij}^{m} + \varepsilon}{s_{ij}^{w} + \varepsilon}\right) \tag{11}$$

We can also use the s_{ij}^{m} and s_{ij}^{w} to calculate the M-value.

If control cell data are available, the RR, OR, and TCR can be calculated as follows:

RR (Risk Ratio)

The RR represents the ratio of the Beta-value in test cells to that in control cells. The formula is:

$$RR_{ij}^{s} = \log_2\left(\frac{\dfrac{s_{ij}^{m} + \varepsilon}{s_{ij}^{m} + s_{ij}^{w} + \varepsilon}}{\dfrac{\sum_{k=1}^{N_c} C_{ik}^{m}/\hat{e}_k + \varepsilon}{\sum_{k=1}^{N_c} C_{ik}^{m}/\hat{e}_k + \sum_{k=1}^{N_c} C_{ik}^{w}/\hat{e}_k + \varepsilon}}\right) \tag{12}$$

Here, N_c is the number of control cells. We only use the raw, non-normalized read counts for the control cells, and only the control cells need to calculate the size factors.

OR (Odds Ratio)

The OR represents the ratio of the M-value in test cells to that in control cells. The formula is:

$$OR_{ij}^{s} = \log_2\left(\frac{\dfrac{s_{ij}^{m} + \varepsilon}{s_{ij}^{w} + \varepsilon}}{\dfrac{\sum_{k=1}^{N_c} C_{ik}^{m}/\hat{e}_k + \varepsilon}{\sum_{k=1}^{N_c} C_{ik}^{w}/\hat{e}_k + \varepsilon}}\right) \tag{13}$$

TCR (Target-Controlled Ratio)

The TCR represents the difference between the Beta-value in test cells and the Beta-value in control cells. The formula is:

$$TCR_{ij}^{s} = \max\left(\frac{s_{ij}^{m}}{s_{ij}^{m} + s_{ij}^{w} + \varepsilon} - \frac{\sum_{k=1}^{N_c} C_{ik}^{m}/\hat{e}_k + \varepsilon}{\sum_{k=1}^{N_c} C_{ik}^{m}/\hat{e}_k + \sum_{k=1}^{N_c} C_{ik}^{w}/\hat{e}_k + \varepsilon}, 0\right) \tag{14}$$

Here, ε is a hyper-parameter to prevent division by zero or undefined values. Using these formulas, we can predict the metrics of methylation levels.

Diffusion start & end point

To determine the probability distribution of the end points in a diffusion process, we calculate the stationary probability based on the combined transition probability T_{ij}. The stationary probability provides insight into the likelihood of a cell ending up in a particular state after many transitions. The formula is:

$$A_j = \frac{\sum_i \left(T_{ij}\right)^n}{\sum_{ij} \left(T_{ij}\right)^n} \tag{15}$$

Here, n represents the number of transitions or changes the cell undergoes, and A_j denotes the probability distribution of the end points. This approach helps us understand the steady-state behavior of cells as they undergo dynamic changes, highlighting the most probable states they will occupy over time.

To calculate the probability distribution of the start points, we adjust our calculations by replacing T_{ij} with its transpose matrix. The formula is:

$$B_{ij} = \frac{\sum_i \left(\left(\frac{T_{ij}}{\sum_i (T_{ij})} \right)^{\mathsf{T}} \right)^n}{\sum_{ij} \left(\left(\frac{T_{ij}}{\sum_i (T_{ij})} \right)^{\mathsf{T}} \right)^n} \quad (16)$$

In this context, B_{ij} denotes the probability distribution of the start points, allowing us to trace the origins of the diffusion process, providing a complementary perspective to the end point distribution.

Theoretical Basis for Transcriptional Impact Analysis

To elucidate the mechanistic relationship between RNA methylation and transcriptional regulation, we developed a computational framework that integrates kinetic modeling of splicing dynamics with methylation state analysis, as illustrated in **Supplementary Figure 4A**. The expression dynamics of individual transcripts are quantified through the temporal derivative of post-splicing RNA (Δs_{ij}), which serves as a proxy for transcriptional output. This differential expression metric is computed through two alternative approaches: a composite measure combining methylation-specific changes ($\Delta s_{ij}^m + \Delta s_{ij}^w$), or through direct kinetic estimation derived from coupled pre-splicing and post-splicing RNA balance equations.

The regulatory influence of methylation is captured through combined methylation levels across transcriptional stages $m_{ij} = m_{ij}^u + m_{ij}^s$. We first employ Spearman or Pearson correlation to evaluate the global relationship between methylation and expression dynamics. The functional relationship is then formalized through a multivariate regression framework:

$$\Delta s_{ij}^m \sim \beta_0 + \beta_1 m_{ij}^u + \beta_2 m_{ij}^s \quad (17)$$

where the partial regression coefficients (β_1, β_2) quantify the stage-specific regulatory effects of methylation on transcriptional output.

Mechanism insights into the formation of pre-splicing- and post-splicing-prominent sites

Method: Categorization of pre-splicing- and post-splicing-prominent m^{6}A sites

To explore the regulatory role of m^{6}A in RNA splicing, we integrated multiple analyses to identify sites with distinct methylation patterns between pre-splicing and post-splicing transcripts. Sites were classified as: (1) Pre-splicing-prominent m^{6}A sites: from the 37,199 high-confidence sites, meeting (i) Median SigRM p-value ≤ 0.05 for pre-splicing RNA; (ii) Absolute meta log2 odds ratio difference ≥ 1. (2) Post-splicing-prominent m^{6}A sites: from the 37,199 high-confidence sites, meeting (i) Median SigRM p-value ≤ 0.05 for post-splicing RNA; (ii) Absolute meta log2 odds ratio difference ≥ 1. (3) All m^{6}A sites: from the full 450,974 sites, meeting (i) Median SigRM p-value ≤ 0.05 for overall RNA; (ii) not meeting the criteria for either pre-splicing- or post-splicing-prominent m^{6}A sites. We then visualized the genomic distribution of the top 500 sites (ranked by lowest p-value) from each category—pre-splicing-prominent, post-splicing-prominent, and all m^{6}A sites—using MetaTX [4].

Method: Categorization of pre- and post-splicing-prominent A-to-I RNA editing sites

To investigate the role of A-to-I RNA editing in splicing regulation, we integrated multiple analyses to identify sites with distinct modification patterns between pre-splicing and post-splicing transcripts. Since A-to-I editing lacks control data, we defined significant sites as those with an absolute meta log2 odds ratio difference ≥ 1 between conditions. Sites were classified as: (1) Pre-splicing-prominent A-to-I editing sites if meta log2 M-value difference ≥ 1 across pre-splicing RNA; (2) Post-splicing-prominent A-to-I editing sites if meta log2 M-value difference ≥ 1 across post-splicing RNA; (3) All A-to-I editing sites if (i) meta log2 M-value difference ≥ 1 across overall RNA; (ii) not meeting the criteria for either pre-splicing- or post-splicing-prominent A-to-I editing sites. We then visualized the genomic distribution of the top 500 sites (ranked by highest M-value) from each category—pre-splicing-prominent, post-splicing-prominent, and other A-to-I editing sites—using MetaTX [4]

Results: pre- and post-splicing-prominent m^{6}A sites

We identified two categories of m^6A sites based on their prominence on pre-splicing or post-splicing RNAs, i.e., the pre-splicing-prominent m^6A sites (with significantly higher methylation level on pre-splicing RNA than post-splicing RNA) and the post-splicing-prominent m^6A sites (with significantly higher methylation level on post-splicing RNA than pre-splicing RNA) (see the ***Categorization of pre- and post-splicing-prominent m⁶A sites*** section for details) and further investigate their transcriptome distribution with MetaTX [4] to analyze their positional enrichment and associated transcript landmarks. As shown in **Supplementary Figure 1D**, the pre-splicing-prominent m^6A sites are more enriched at the 3' side of CDS. In contrast, the post-splicing-prominent sites exhibited a pronounced enrichment around the start codon and on the 5' end of CDS. The distinct distribution pattern of the two strongly implies functional and regulatory difference.

There exist several mechanisms that could explain the increased m^6A level on pre-splicing RNAs than post-splicing RNAs for pre-splicing-prominent m^6A sites. One possibility is that m^6A is predominantly deposited before splicing but is actively removed during or after the splicing process. Another possibility is that the m^6A modification at these sites is more stable in pre-splicing RNA compared to post-splicing RNA, leading to higher retention of the modification before splicing [5,6]. Additionally, the presence of m^6A at these sites may interfere with the binding of splicing factors to pre-splicing RNA, potentially influencing the splicing event [7,8]. Previous studies have linked IDH1 [9], and CTCF [10] to both m^6A regulation and splicing, supporting the functional interplay between RNA modifications and splicing machinery. Recent studies demonstrated that m^6A deposited within the CDS of post-splicing RNAs could directly influence translation by inducing ribosome stalling and triggering mRNA degradation [11]. This translation-coupled decay was exemplified by a YTHDF2–DCP2-dependent pathway [12], and by ribosome collision-triggered decay at m6A-modified sites [13]. The observed decreased m^6A level on post-splicing RNA than pre-splicing RNA could also therefore be a direct consequence of this mechanism. These diverse possible mechanisms highlight the dynamic and regulated role of m^6A in RNA processing, with m^6A deposition and removal being closely linked to splicing events and pre-splicing RNA maturation [5]. Among the 10,963 pre-splicing-prominent sites, we identified 21 sites that exclusively show methylation read counts in pre-splicing RNA but with no methylation signals detected from post-splicing RNA at all, which represents the most extreme scenario (detailed in **Supplementary Table 2**).

In contrast, sites showing higher methylation level in post-splicing RNA than pre-splicing RNA may suggest alternative mechanisms. The simplest mechanism is that m^6A is further installed during or after the splicing process [14]. One explanation is that the splicing process itself may create a structural environment that makes the site more accessible or more stable for methylation [8,15]. For example, the m^6A at the 5′ end may enhance transcript stability by protecting against decapping enzymes (e.g., DCP1/2) [16] or 5'→3' exonucleases (e.g., XRN1) [17], which can lead to the observed pattern. Additionally, compared to pre-splicing RNA, post-splicing RNA may be less susceptible to degradation or demethylation, allowing m^6A marks to be more stably retained [18]. Furthermore, m^6A modification may also play a role in regulating splicing efficiency [19]; if methylation at a given site promotes the splicing process, this could explain the higher methylation levels observed in post-splicing RNA. Among the 588 post-splicing-prominent sites, we identified 32 m^6A sites that exclusively show methylation signals in post-splicing RNAs only, strongly suggesting that these sites are methylated during or after splicing (detailed in **Supplementary Table 2**).

Results: pre- and post-splicing-prominent A-to-I editing sites

chromaffin cell differentiation dataset

Pre-splicing- and post-splicing-prominent A-to-I RNA editing sites were identified (see ***Methods: Categorization of pre- and post-splicing-prominent A-to-I RNA editing sites***), resulting in 445 and 442 sites, respectively. Pre-splicing-prominent sites may reflect several regulatory mechanisms: the modification could be more stable or preferentially retained in pre-splicing RNA, leading to reduced signals in post-splicing transcripts [20]; or A-to-I editing might modulate splicing by affecting factor binding or splice site selection [21]. Among the 445 pre-splicing candidate sites, 41 sites (**Supplementary Table 3**) were detected exclusively in pre-splicing RNA, representing the strongest preference [22].

Post-splicing-prominent sites likely arise during or after splicing. Splicing may create structural contexts that enhance accessibility or stability of specific sites [23,24], or post-splicing RNA may be less susceptible to degradation, allowing edits to persist [25,26]. A-to-I editing could also influence splicing efficiency,

explaining higher editing levels in post-splicing RNA [21]. Among 442 post-splicing sites, 255 (**Supplementary Table 3**) were detected exclusively in post-splicing RNA, consistent with editing during or after splicing, especially near splice junctions [25-27]. Nevertheless, low read coverage may contribute to false positives.

MetaTX [4] analysis of transcriptome distribution showed that pre-splicing-prominent sites were slightly enriched around the start codon, whereas post-splicing-prominent sites were pronouncedly enriched in the 5′ UTR (**Supplementary Figure 1E**).

mESC embryoid body (EB) differentiation dataset

Pre-splicing- and post-splicing-prominent A-to-I RNA editing sites were identified (see *Methods: Categorization of pre-splicing and post-splicing-prominent A-to-I RNA editing sites*), yielding 805 and 855 sites, respectively. Mechanistic interpretations are similar to the chromaffin dataset. Among 805 pre-splicing sites, 81 (**Supplementary Table 4**) were detected exclusively in pre-splicing RNA. Among 855 post-splicing sites, 428 (**Supplementary Table 4**) were detected exclusively in post-splicing RNA, suggesting editing predominantly occurs during or after splicing, particularly near splice junctions [25-27]. However, given the small number of reads in the A-to-I editing sites, false positives can not be entirely ruled out.

MetaTX [4] analysis indicated that both pre-splicing- and post-splicing-prominent sites showed enrichment toward the end of the 5′ UTR (**Supplementary Figure 1F**).

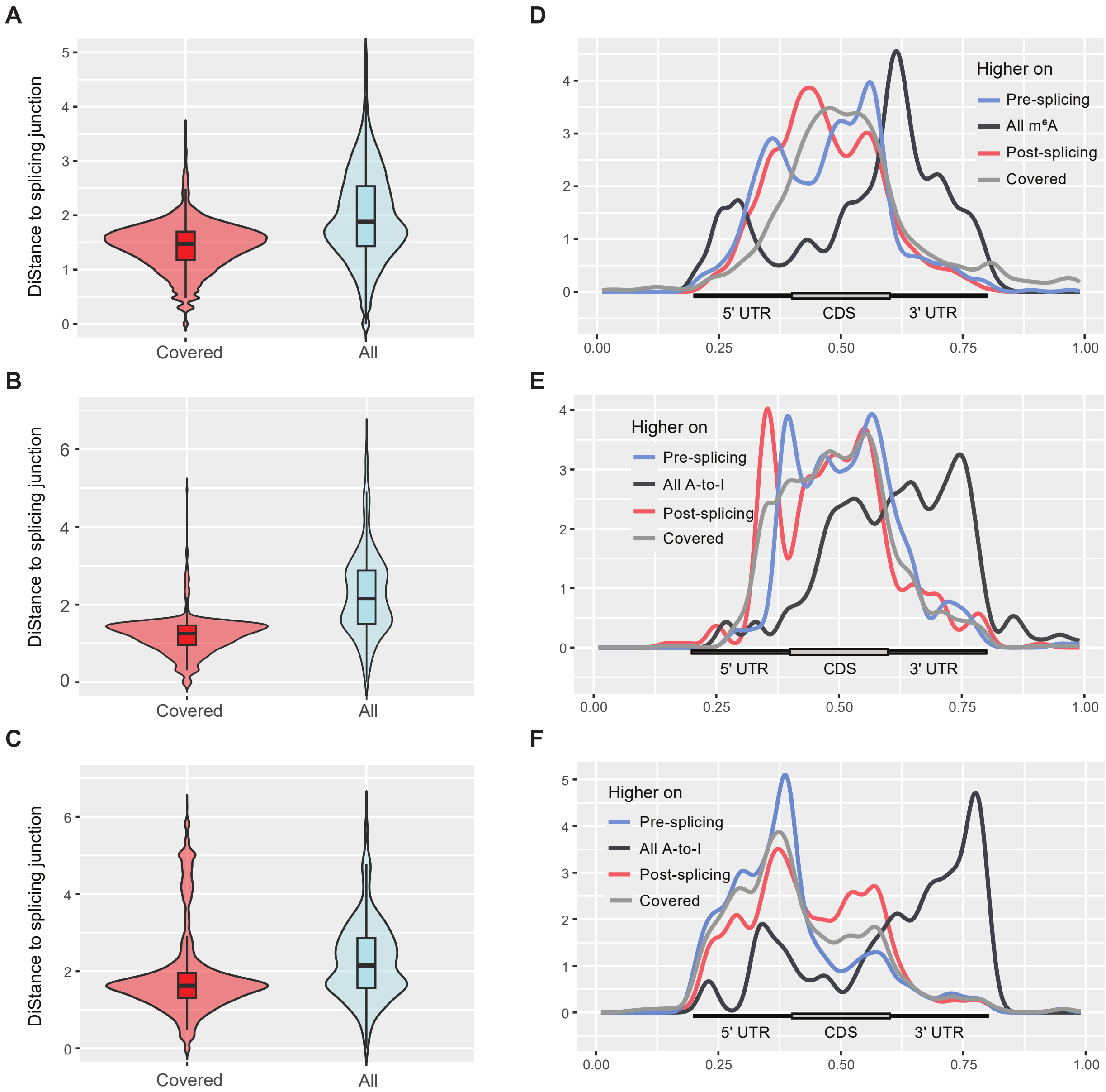

Supplementary Figure 1. Transcriptomic Distribution of m^6A and A-to-I Modification Sites Across Pre- and post-splicing RNA. A. Distance distribution of 37,199 scDART-seq m^6A candidate sites detected on both pre-splicing and post-splicing RNAs, compared to randomly selected exonic sites. The reliance on splice-aware annotation and joint coverage restricts the analysis to splice-junction–proximal regions. **B.** Distance distribution of A-to-I editing sites relative to the nearest splice junction in the chromaffin cell differentiation dataset, compared with random exonic sites, indicating the analysis is limited to splice-junction–proximal regions. **C.** Distance distribution of A-to-I editing sites relative to the nearest splice junction in the mESC embryoid body (EB) differentiation dataset, compared to random exonic sites, again showing restriction to junction-proximal regions. **D.** Transcriptome-wide distribution of m^6A sites visualized using MetaTX. Pre-splicing-prominent sites show enrichment on the 3′ side of the CDS, whereas post-splicing-prominent sites are enriched near the start codon and on the 5′ side of the CDS. Distributions for all m^6A sites and analyzed regions are also shown. **E.** Transcriptome distribution of A-to-I editing sites (pre-splicing-prominent, post-splicing-prominent, and all sites) visualized by MetaTX in the chromaffin cell differentiation dataset. Pre-splicing-prominent sites show mild enrichment near the start codon, whereas post-splicing-prominent sites are enriched in the 5′ UTR. **F.** Transcriptome distribution of A-to-I editing sites

in the mESC EB dataset. Both pre-splicing- and post-splicing-prominent sites show enrichment toward the end of the 5′ UTR.

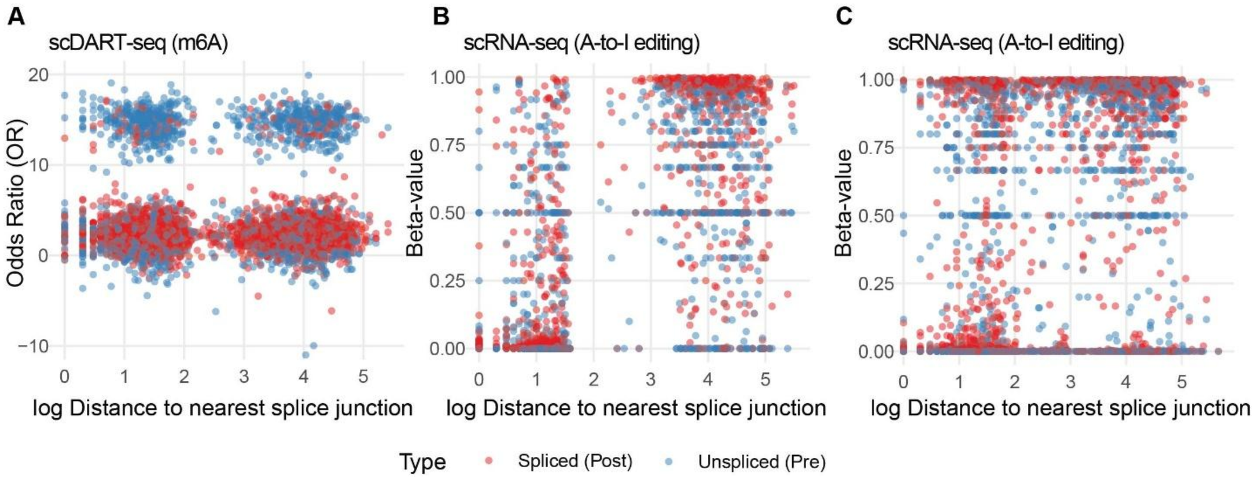

Supplementary Figure 2. Relationship between distance to splice junctions and RNA modification levels in pre- and post-splicing transcripts. A. Distribution of 37,199 scDART-seq–identified m6A candidate sites (detected in both pre-splicing and post-splicing RNAs) as a function of distance to the nearest splice junction. Modification levels are quantified as odds ratios (ORs). **B.** Distribution of A-to-I editing sites relative to the nearest splice junction in both pre-splicing and post-splicing RNAs in the chromaffin cell differentiation dataset. Editing levels are quantified using Beta-value**. C.** Same analysis as in (B), performed on the mESC embryoid body (EB) differentiation dataset.

Comparison of the pre- and post-splicing m^6A epitranscriptomes from DART-seq and MeRIP-seq

Method: Comparison of the pre- and post-splicing m^{6}A epitranscriptomes from MeRIP-seq

Raw sequencing data were obtained from the GEO database (MeRIP-seq: GSE29714 [28]) and aligned to the human hg38 reference genome using STAR [29]. Reads were annotated to distinguish unspliced (pre-splicing) and spliced (post-splicing) transcripts, and two independent BAM files were generated for each sample corresponding to pre-splicing and post-splicing RNAs

To quantify single-nucleotide–resolution methylation, we first used the m^{6}A candidate sites identified from the scDART-seq dataset and extracted read counts from both pre-splicing and post-splicing MeRIP-seq BAM files. For each site, we obtained methylated and unmethylated read counts across the four MeRIP-seq samples (two controls and two tests), resulting in two biological replicates for each of the following categories: pre-methylated, pre-unmethylated, post-methylated, and post-unmethylated.

Differential methylation between pre-splicing and post-splicing RNAs was assessed using DESeq2 [30]. Sites with an absolute $\log_2$ fold change ($|\log_2 FC| > 1$) and a p-value ≤ 0.05 were defined as differentially methylated. In parallel, we also applied Fisher's exact test to evaluate differential methylation by aggregating replicate read counts for each condition. The same significance criteria ($|\log_2 FC| > 1$ and $p \leq 0.05$) were used to identify differentially methylated sites.

Results: Comparison of the pre- and post-splicing m^{6}A from MeRIP-seq

To validate our observation that both DART-seq and scDART-seq consistently reveal higher m6A levels in pre-splicing RNAs, we examined whether MeRIP-seq data showed similar trends at the same candidate sites. Consistent with the DART-derived datasets, DESeq2-based differential methylation analysis (**Supplementary Figure 3F**) identified more hypermethylated sites in pre-splicing RNAs (167 sites) compared with post-splicing RNAs (133 sites). Fisher's exact test yielded concordant results (**Supplementary Figure 3G**), identifying 377 pre-splicing–enriched sites versus 214 post-splicing–enriched

sites. Together, these results support the conclusion that pre-splicing RNAs harbor stronger m6A modification signals than post-splicing RNAs across independent profiling platforms.

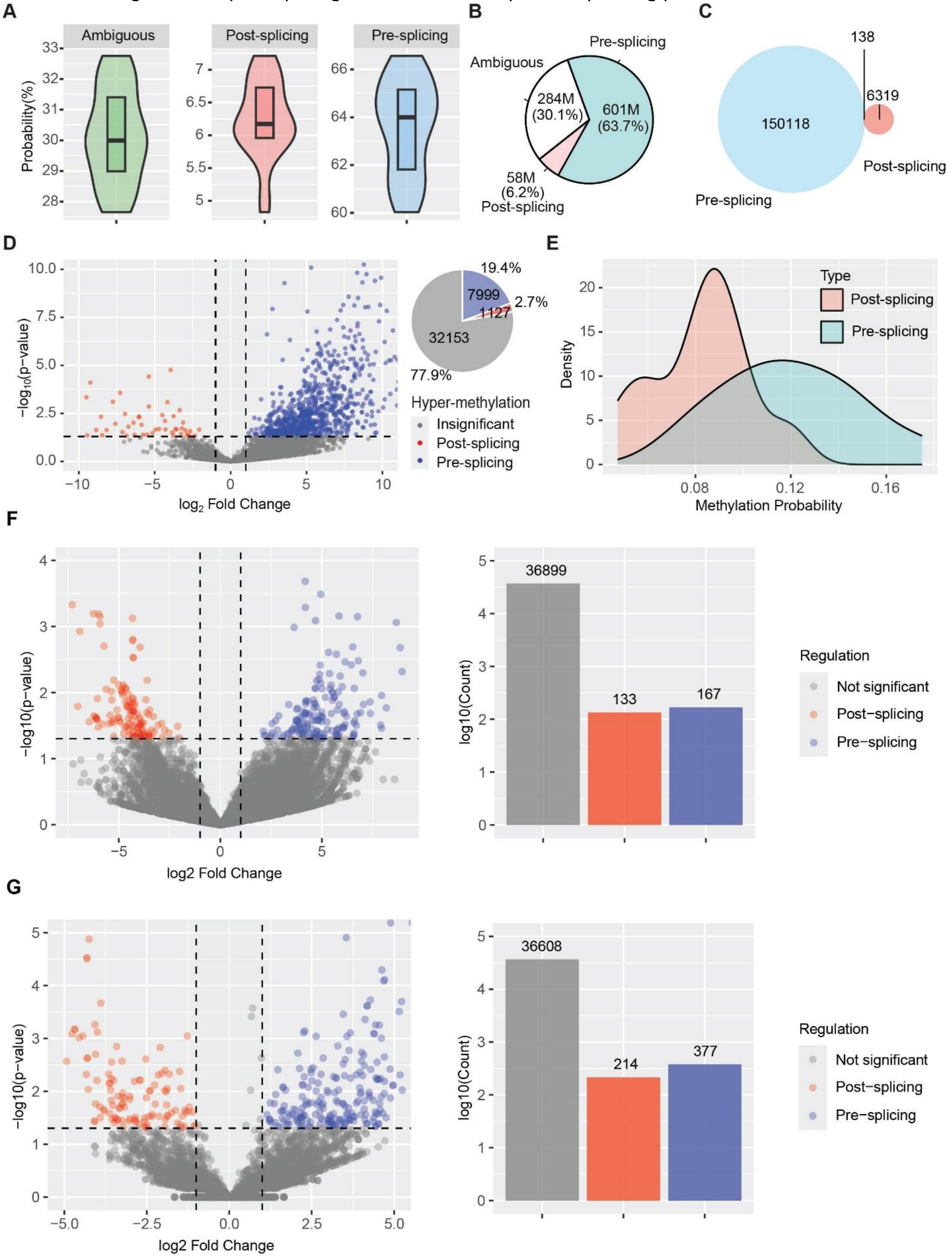

Supplementary Figure 3. Differential m6A methylation between pre- and post-splicing RNAs. A–E, another DART-seq. A. Proportions of pre-splicing, post-splicing, and ambiguous RNA reads vary across individual cells in the DART-seq dataset analyzed. **B.** Aggregated across all cells, 6.2% of reads (58 million) are confidently assigned to post-splicing RNAs, 63.7% (601 million) to pre-splicing RNAs, and the remaining 30.1% (284 million) are ambiguous, lacking clear classification. **C.** Using DESeq2, 150,118 m^6A sites were detected exclusively in pre-splicing RNAs, whereas 6,319 sites were found only in post-splicing RNAs, providing a first glimpse into the distinct landscapes of the pre-splicing and post-splicing epitranscriptomes. **D.** Volcano plot showing differential m^6A methylation between pre-splicing and post-splicing RNAs. 7,999 sites are significantly hypermethylated in pre-splicing RNAs, whereas 1,127 sites are hypermethylated in post-splicing RNAs. **E.** Density distribution of methylation proportions for pre-splicing and post-splicing RNAs in DART-seq (aggregated across 11 replicates), showing higher methylation levels in pre-splicing RNAs. **F–G, MeRIP-seq. F.** Differential methylation analysis using DESeq2 for m^6A candidate sites extracted from pre-splicing and post-splicing MeRIP-seq BAM files. Each dot represents a single nucleotide site. Sites with $|\log_2 FC| > 1$ and $p \le 0.05$ are highlighted. A total of 167 sites were significantly hypermethylated in pre-splicing RNAs, whereas 133 sites were enriched in post-splicing RNAs. **G.** Fisher's exact test performed on aggregated replicate counts showing a consistent trend. Using the same significance criteria ($|\log_2 FC| > 1$ and $p \le 0.05$), 377 sites were identified as pre-splicing–enriched and 214 as post-splicing–enriched.

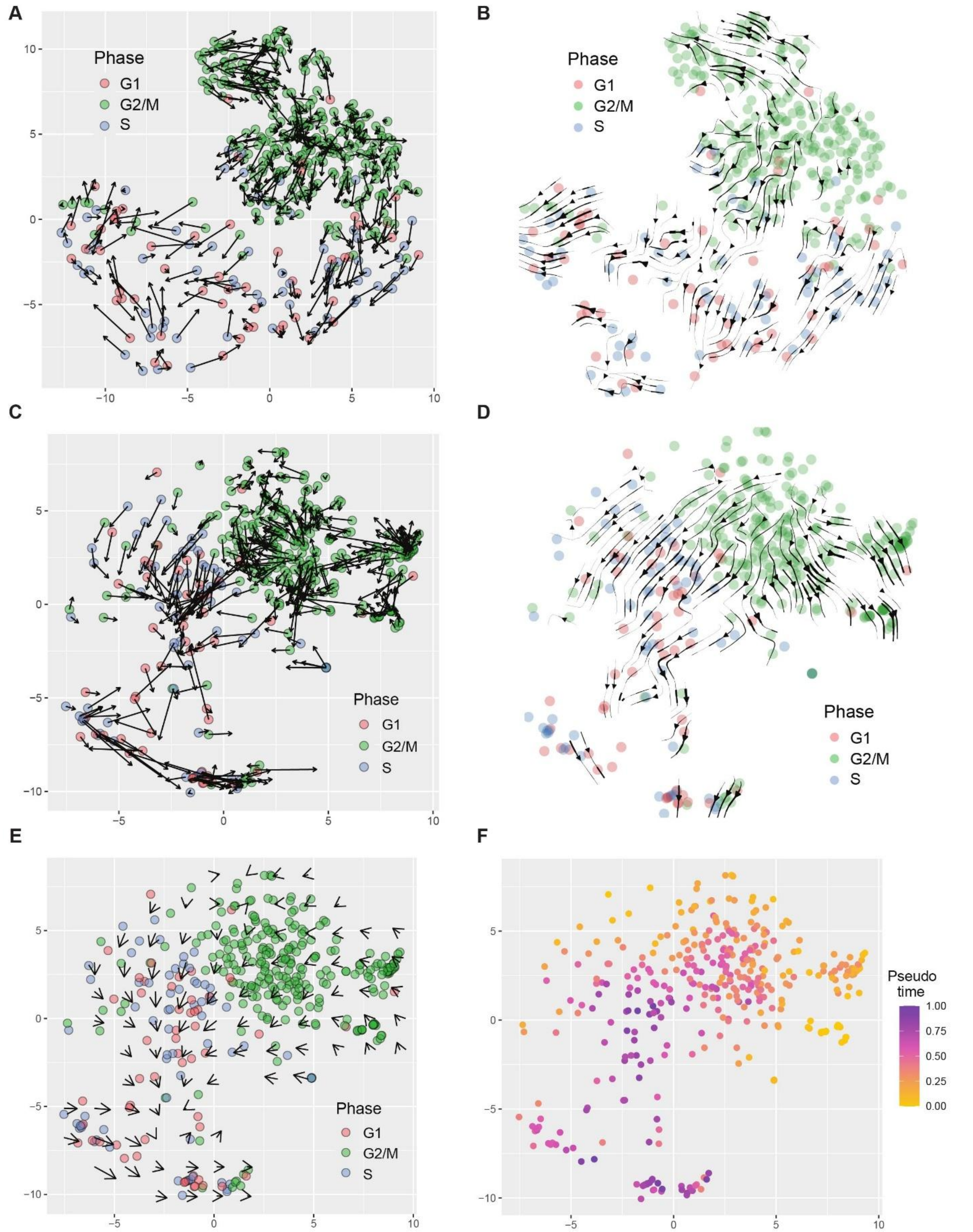

Supplementary Figure 4. Visualization of Epitranscriptomic Dynamics and Methylation Trajectories from scDART-seq Data. A. Single-cell m^{6}A epitranscriptomic velocity projected onto a UMAP embedding. **B**. Single-cell RNA velocity projected onto the UMAP embedding using scVelo. **C.** Single-cell m^{6}A epitranscriptomic velocity projected onto a t-SNE embedding. **D.** Single-cell RNA velocity projected onto

the t-SNE embedding using scVelo. **E.** Velocity field illustrating the overall dynamic landscape of m^6A RNA modifications using t-SNE. **F.** Pseudotime estimation of cell-cycle progression derived from the m^6A-informed transition matrix, ranging from 0 (start) to 1 (end), capturing continuous cell-cycle dynamics in t-SNE space.

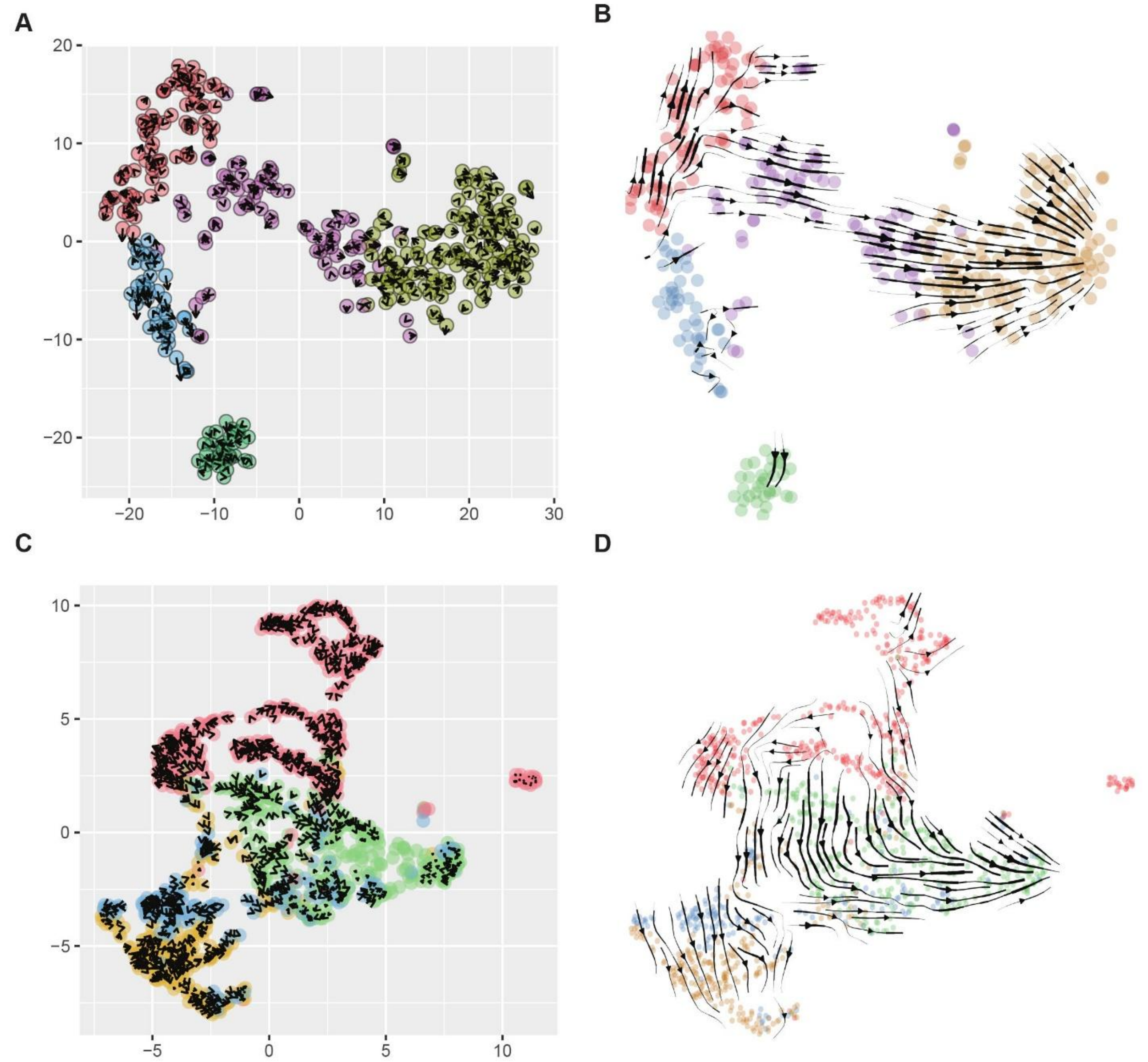

Supplementary Figure 5. Visualization of Epitranscriptomic Dynamics and Modification Trajectories from scRNA-seq Data. A. Single-cell A-to-I editome velocity in the chromaffin cell differentiation dataset. **B.** Single-cell RNA velocity in the chromaffin cell differentiation dataset using scVelo. **C.** Single-cell A-to-I editome velocity in the mESC embryoid body (EB) differentiation dataset. **D.** Single-cell RNA velocity in the mESC EB differentiation dataset using scVelo.

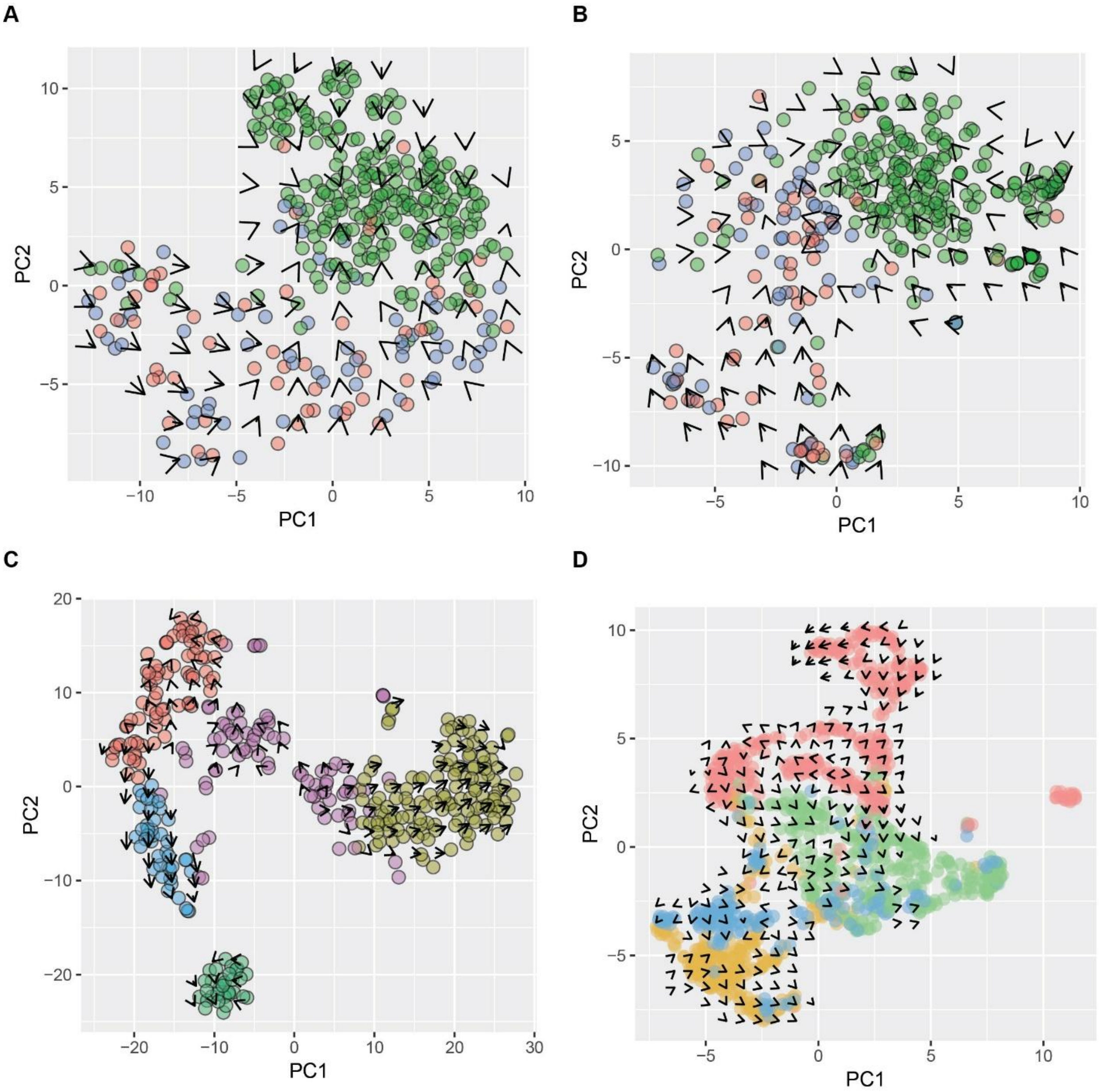

Supplementary Figure 6. Visualization of Epitranscriptomic Dynamics and Modification Trajectories using all sites. A. Single-cell m^{6}A epitranscriptomic velocity inferred from all 36,739 sites, projected onto a UMAP embedding. **B.** Single-cell m^{6}A epitranscriptomic velocity inferred from all 37,199 sites, projected onto a t-SNE embedding. **C.** Single-cell A-to-I editome velocity inferred from all 189 sites in the chromaffin cell differentiation dataset. **D.** Single-cell A-to-I editome velocity inferred from all 217 sites in the mESC embryoid body (EB) differentiation dataset.

Method: Evaluation of the effectiveness of our site selection strategy

Trajectory-based Evaluation for Cyclic Processes (A-B)

For the cyclic cell-cycle data (scDART-seq m6A), we utilized Cosine Similarity rather than Spearman correlation for evaluation. While high Spearman correlation can reflect linear ordering, it fails to distinguish between a simple linear path and a closed loop (cyclicity). To verify if the inferred velocities maintain a complete biological circuit, we focused on two critical transition boundaries:

1. Boundary Identification: We selected G1/S cells positioned near the G2/M transition and G2/M cells located at the terminal end (furthest from the G1/S origin).
2. Vector Assessment: We calculated the cosine similarity between the predicted transition vector (derived as the probability-weighted displacement toward neighbors) and an "ideal" vector (pointing toward the centroid of the target cell cluster).
3. Cyclicity Validation: Performance was quantified by whether G1/S boundary cells correctly pointed toward G2/M and whether terminal G2/M cells correctly pointed back toward G1/S. This ensures the model captures closed-loop dynamics rather than an incomplete linear progression.

Pseudotime-based Evaluation for Unidirectional Differentiation (C-D)

For unidirectional processes (A-to-I editome and mESC differentiation), we evaluated performance using the Spearman rank correlation between inferred pseudotime and known developmental stages.

1. Pseudotime Inference: For VeloRM, pseudotime was derived from the diffusion density of the transition matrix; for scVelo, the internal latent time was used.
2. Metric Calculation: Annotated cell stages were mapped to a numerical sequence reflecting the biological lineage (e.g., Stage 0-1-2). A higher correlation coefficient indicates that the inferred pseudotime ordering more accurately preserves the ground-truth developmental progression.

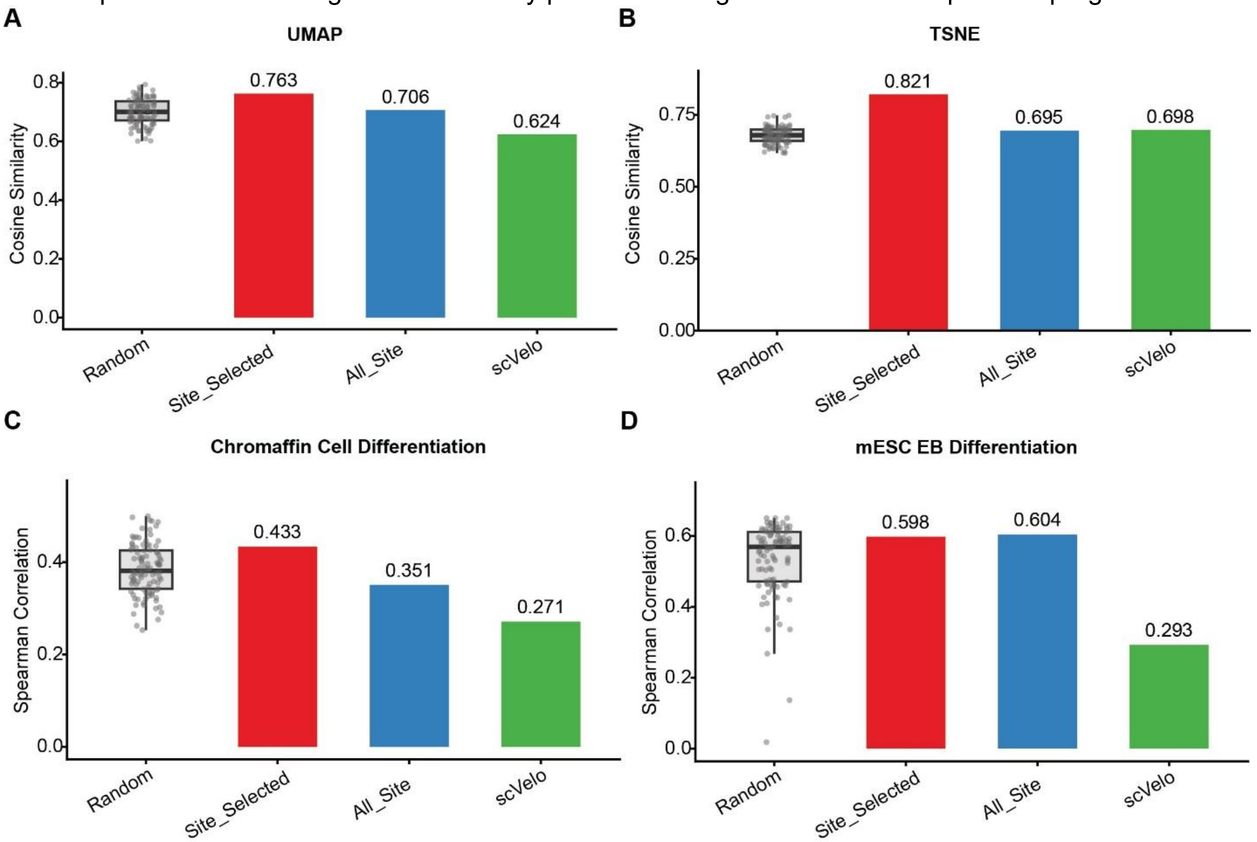

Supplementary Figure 7. Comparison of site selection strategies using trajectory-based and pseudotime-based evaluations. A-B. Single-cell m6A epitranscriptomic velocity inferred from scDART-seq data, comparing site selection based on our strategy versus randomly selected sites. We quantified the cosine similarity between the predicted transition direction, derived from the transition probability matrix, and the expected direction toward target cell states in the embedding space. Empirical p-values for the selected sites relative to random selections were 0.0792 for UMAP and 0.0099 for t-SNE. **C.** Single-cell A-to-I editome velocity in the chromaffin cell differentiation dataset. As this represents a unidirectional differentiation process, performance was evaluated by the Spearman correlation between inferred pseudotime and annotated cell states. Empirical p-values for the selected sites relative to random selections were 0.0792 for UMAP and 0.0099 for t-SNE. **D.** Same as in (C), shown for the mESC embryoid body differentiation dataset. The empirical p-value was 0.317.

Transcriptional Impact Analysis

Method: Transcriptional impact - correlation analysis with the velocity of RNAs

To systematically investigate the transcriptional impact of RNA velocity, we developed a computational framework. We first modeled the velocity dynamics of pre-splicing and post-splicing RNA to capture site-level changes in transcript abundance over time. To assess whether RNA modifications enhance or suppress transcription, we calculated Spearman correlations between modification levels and future changes in post-splicing RNA abundance. Sites with an absolute correlation coefficient ≥ 0.2 were considered to exhibit a transcriptionally relevant impact of methylation.

To determine whether the regulatory influence of methylation is primarily driven by modifications in pre-splicing RNA or post-splicing RNA, we performed linear regression using methylation levels from both RNA types as predictors of RNA velocity. For sites where all regression coefficients had p-values ≤ 0.05, we interpreted larger coefficients (in positively correlated models) as indicating stronger regulatory importance, while more negative coefficients were considered more important in negatively correlated models.

To validate the reliability of the linear model, we applied a train-test split. If the root means square error (RMSE) of the test set deviated by no more than ±10% from that of the training set, the model was deemed well-fitted.

Results

To investigate the functional impact of RNA methylation, we developed a framework to explore the relationship between methylation levels in pre-splicing RNA and post-splicing RNA and the subsequent changes in the abundance of post-splicing RNA. Our goal was to determine whether RNA methylation enhances or represses transcription, and whether this regulatory effect is primarily associated with methylation in pre-splicing RNA or post-splicing RNA.

As shown in **Supplementary Figure 8A**, our framework first estimates the future changes in post-splicing RNA levels using pre-splicing RNA and post-splicing RNA read counts, following the framework introduced by [31]. We then correlate these changes with methylation levels and apply linear regression to assess the contribution of pre-splicing RNA and post-splicing RNA methylation, using regression coefficients to evaluate feature importance (see **Supplementary Note: Theoretical Framework and Computational Details— *Theoretical Basis for Transcriptional Impact Analysis***).

We first computed Spearman correlations between methylation levels (pre-splicing RNA, post-splicing RNA, and overall) and the change in post-splicing RNA abundance (Δpost-splicing RNA). As shown in **Supplementary Figure 8B**, methylation level is strongly negatively correlated with Δpost-splicing RNA. Recent studies have similarly found that the presence of m^6A sites increases the decay rate of many cellular RNAs, with RNA decay correlating with m^6A levels in the CDS and occurring in a translation-dependent manner [32]. Using our framework, we identified a total of 3,086 sites with significant correlations to Δpost-splicing RNA. Among these, 10 sites were positively correlated, with 9 primarily influenced by methylation in post-splicing RNA and only 1 by pre-splicing RNA. In contrast, 3,076 sites were negatively correlated, with 3,074 predominantly affected by pre-splicing RNA methylation, while only 2 were influenced by post-splicing RNA methylation (**Supplementary Figure 8C**). These identified sites were further validated using cell subsets, confirming the robustness of the observed correlations (see the ***Transcriptional Impact - Correlation Analysis with the Velocity of RNAs*** section for details). Fisher's exact test revealed a strong association between the direction of the correlation and the methylation source (p-value < 2.2e-16, odds ratio = 11155.21), indicating that transcription-enhancing methylation is primarily associated with post-splicing RNA, while transcription-repressive methylation is predominantly driven by pre-splicing RNA.

This striking divergence likely reflects distinct functional roles of m^6A at different transcript stages. Methylation in post-splicing RNA can promote transcript stability, nuclear export, and translation efficiency, thereby increasing the apparent abundance of post-splicing transcripts [16,33]. In contrast, elevated methylation in pre-splicing RNA may disrupt proper splicing or nuclear processing, ultimately limiting the production of post-splicing transcripts and suppressing their abundance [5,34]. This mechanistic difference explains why positively correlated sites are mostly dominated by post-splicing RNA methylation, while negatively correlated sites are predominantly driven by pre-splicing RNA methylation. **Supplementary**

Figure 8D and **8E** illustrate representative examples: **Supplementary Figure 8D** shows a site where methylation in post-splicing RNA enhances transcription, while **Supplementary Figure 8E** shows a site where methylation in pre-splicing RNA suppresses transcription.

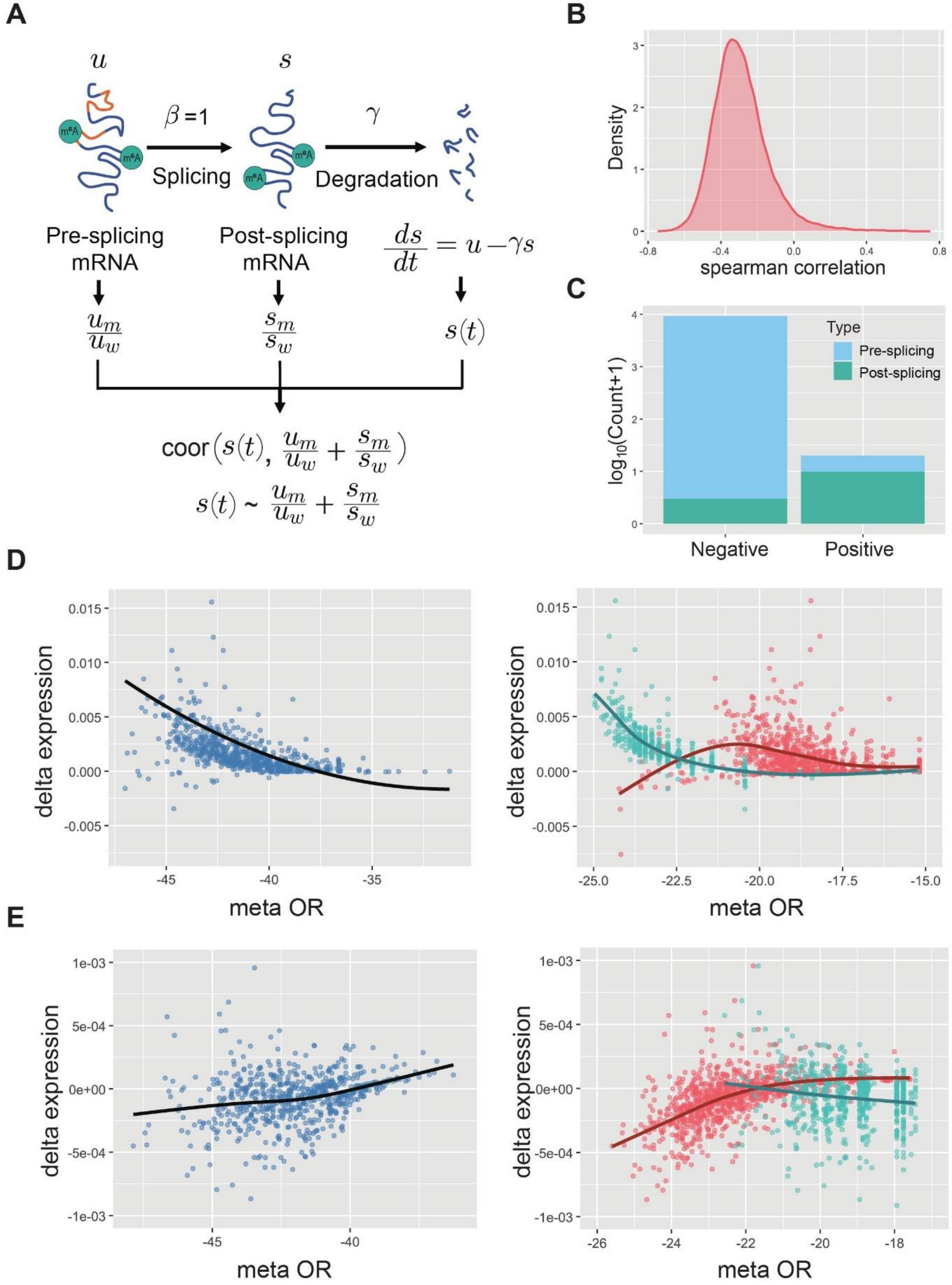

Supplementary Figure 8. Transcriptional impact analysis of RNA methylation. A. Framework to assess the transcriptional impact of methylation by estimating future changes in post-splicing RNA abundance based on pre-splicing and post-splicing read counts, followed by linear regression to quantify contributions from pre-splicing RNA and post-splicing RNA methylation. **B.** Spearman correlation between methylation levels and changes in post-splicing RNA abundance (Δpost-splicing RNA) **C.** Classification of methylation sites based on correlation direction (positive or negative) and the dominant transcript type (pre-splicing RNA or post-splicing RNA) influencing transcriptional change. **D.** A representative site (chr9:100,340,350, TEX10) where methylation in post-splicing RNA is positively associated with increased post-splicing RNA abundance, suggesting enhanced stability or export, shown as an illustrative example. **E**. A representative site (chr3: 184,300,392, PSMD2) where methylation in pre-splicing RNA is negatively associated with post-splicing RNA levels, potentially due to splicing inhibition, shown as an illustrative example.

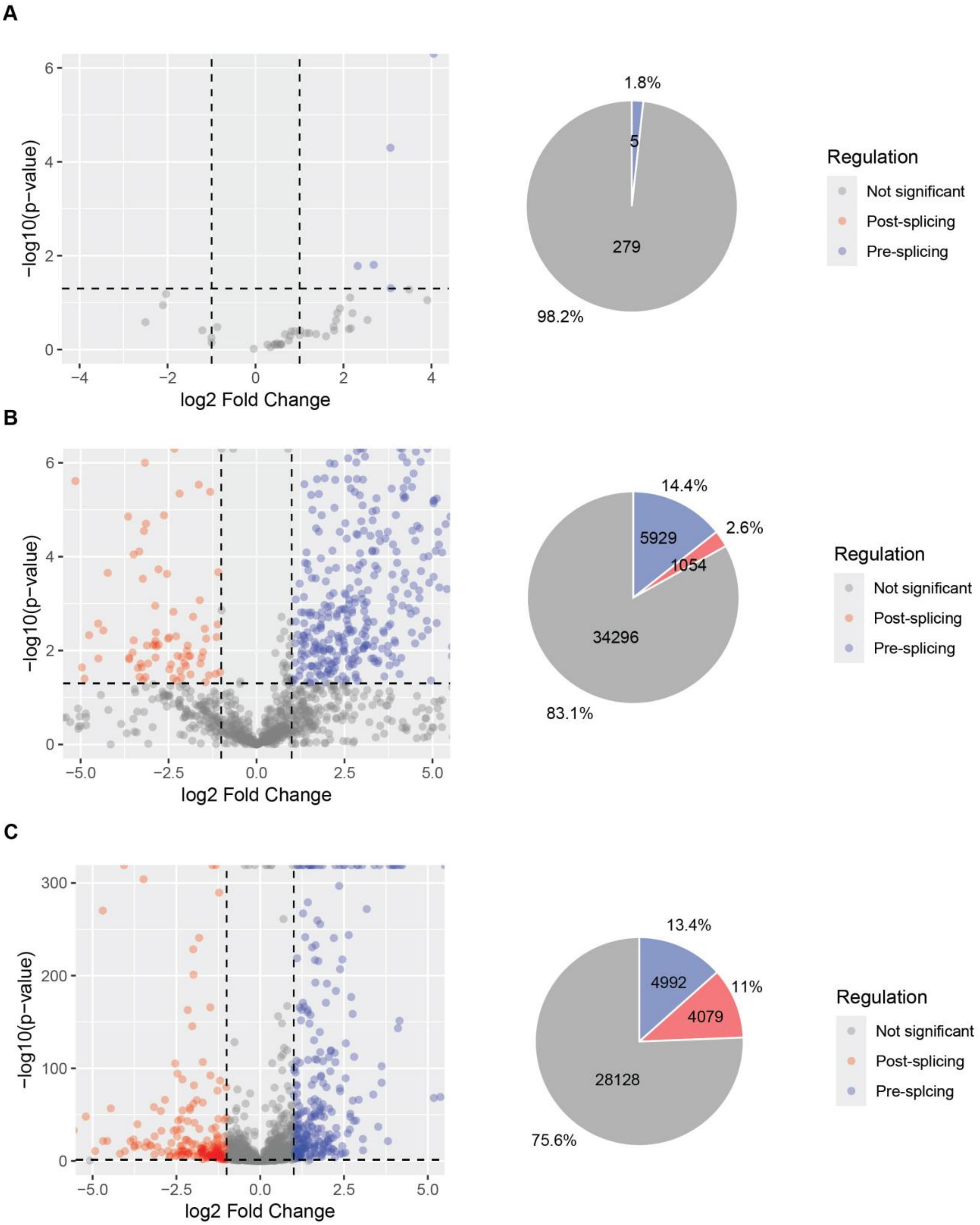

Supplementary Figure 9. Differential m6A methylation analysis using a paired binomial model. To account for the within-cell dependency between pre-splicing and post-splicing reads, we employed a paired binomial test as a robust alternative to models assuming sample independence. This approach explicitly incorporates the pairing structure of reads derived from the same cell to identify differentially methylated sites. **A–C.** Volcano plots illustrating the differential methylation status

between pre-splicing and post-splicing RNAs across three datasets: DART-seq (GSE125780), DART-seq (GSE297551), and scDART-seq (GSE180954). **A.** In GSE125780, 5 sites are significantly hypermethylated in pre-splicing RNAs, while 0 are hypermethylated in post-splicing RNAs. **B.** In GSE297551, 5,929 sites are significantly hypermethylated in pre-splicing RNAs, compared to 1,054 in post-splicing RNAs. **C.** In GSE180954, 4,992 sites show significant hypermethylation in pre-splicing RNAs, versus 4,079 in post-splicing RNAs.

A

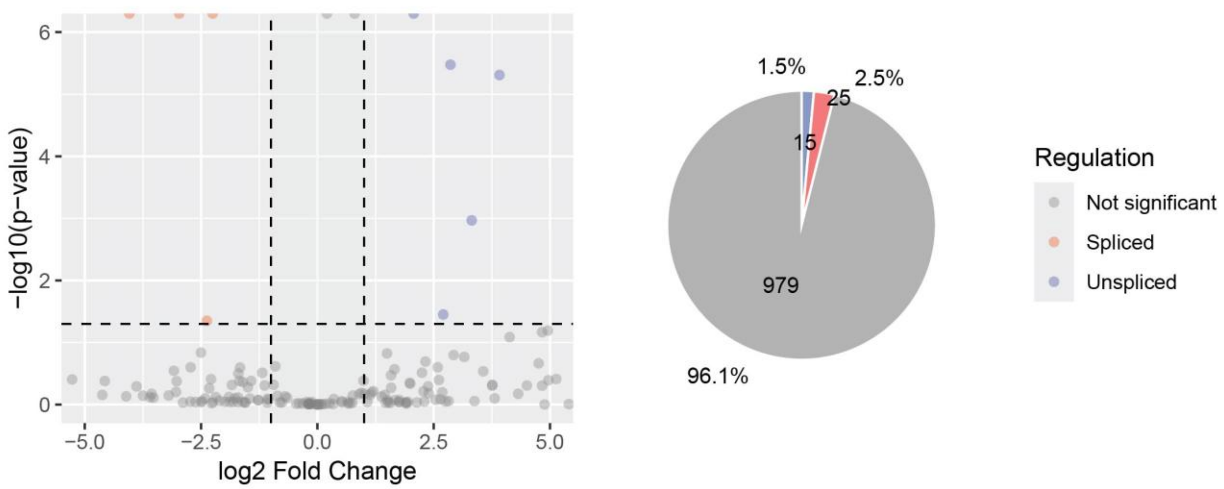

B

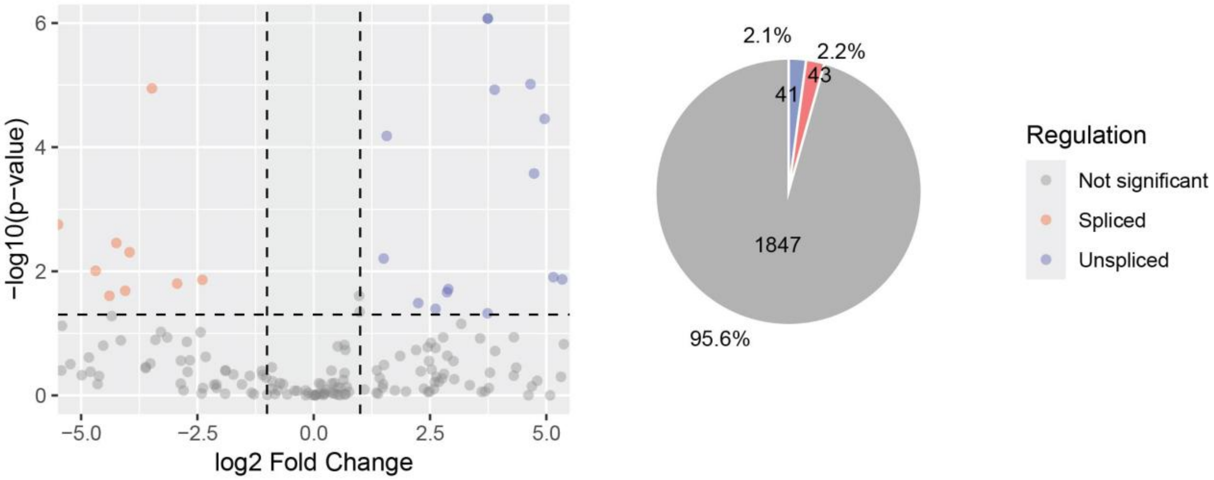

Supplementary Figure 10. Differential A-to-I editing analysis using a paired binomial model. To account for the within-cell dependency between pre-splicing and post-splicing reads in scRNA-seq data, we applied a paired binomial test to identify differentially edited sites. This approach addresses the potential violation of the independence assumption by explicitly incorporating the pairing structure of reads within individual cells. **A–B.** Volcano plots illustrating the differential editing status between pre-splicing and post-splicing RNAs for two scRNA-seq datasets: GSE99933 and GSE151334. **A.** In GSE99933, 15 sites are significantly hyper-edited in pre-splicing RNAs, while 25 are hyper-edited in post-splicing RNAs. **B.** In GSE151334, 41 sites show significant hyper-editing in pre-splicing RNAs, compared to 43 in post-splicing RNAs.

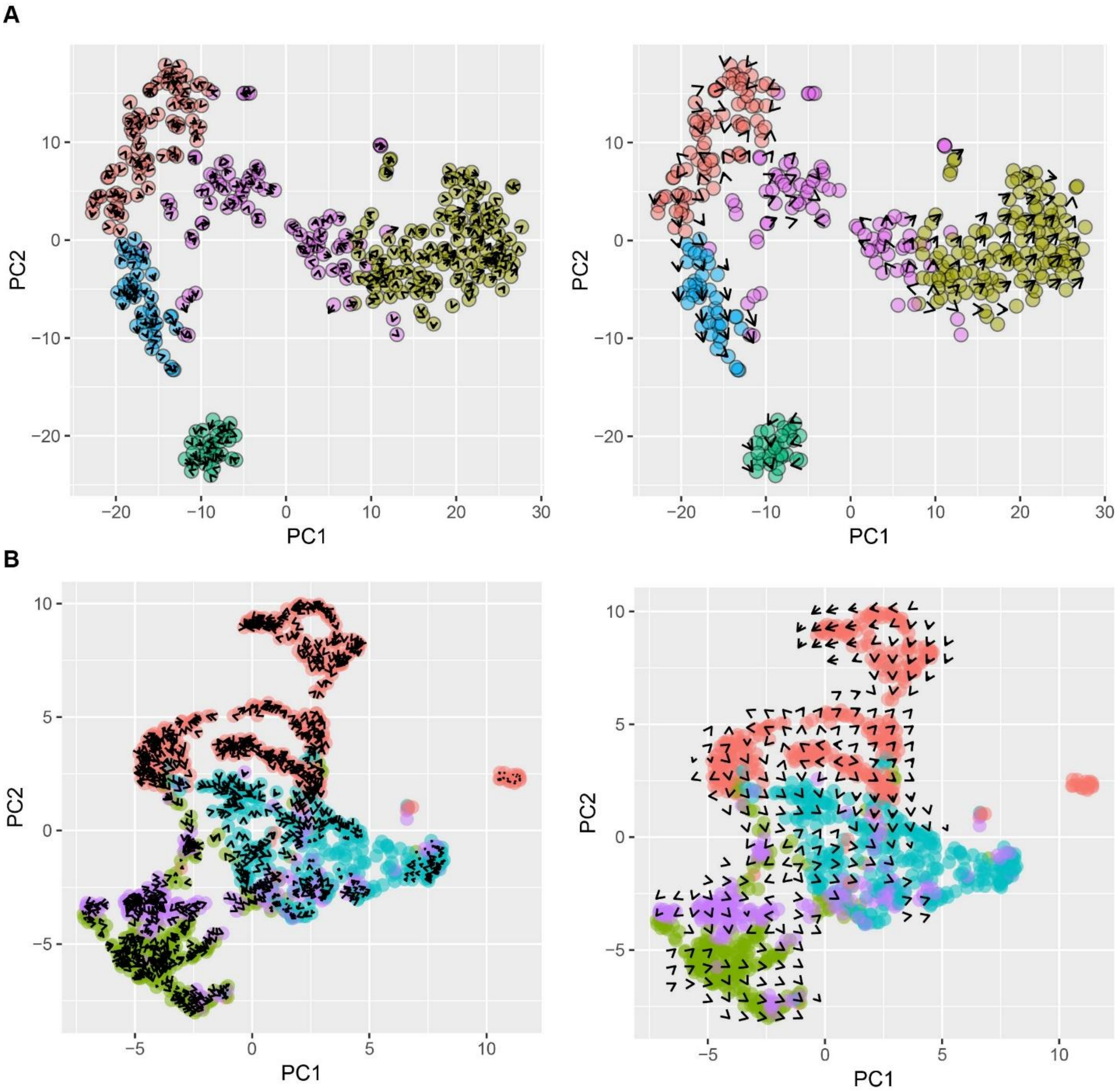

Supplementary Figure 11. Robustness of epitranscriptomic dynamics to the handling of ambiguous reads. A. Single-cell A-to-I editome velocity in the chromaffin cell differentiation dataset, computed after probabilistic reassignment of ambiguous reads, where 50% of ambiguous reads were assigned to the pre-splicing category and the remaining 50% to the post-splicing category. **B.** Single-cell A-to-I editome velocity in the mouse embryonic stem cell (mESC) embryoid body (EB) differentiation dataset under the same reassignment strategy. In both datasets, the inferred velocity fields and differentiation trajectories are highly consistent with those obtained under the default setting (excluding ambiguous reads), indicating that the inferred epitranscriptomic dynamics are robust to the treatment of ambiguous reads.

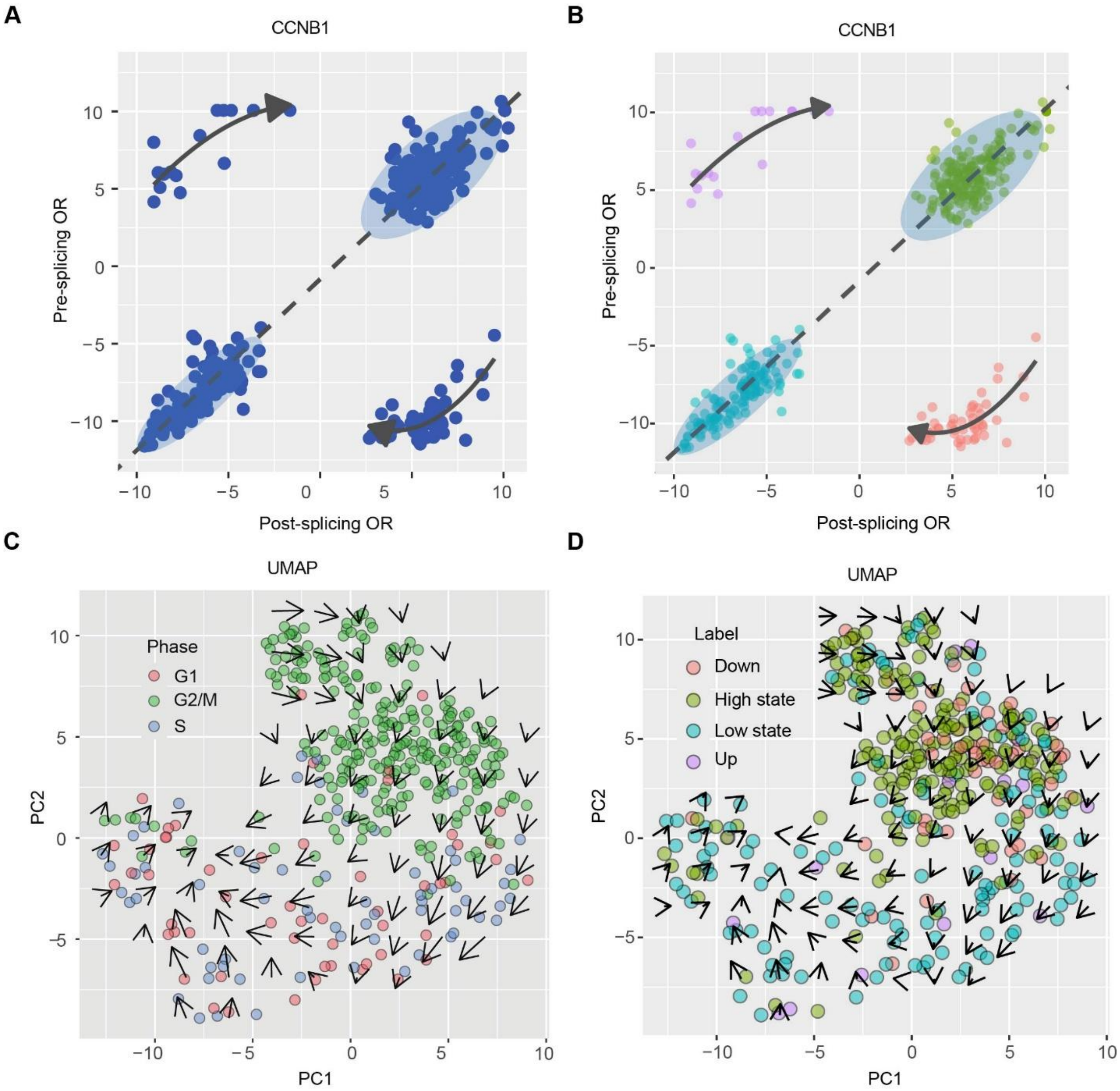

Supplementary Figure 12. Dynamic m^{6}A methylation states across the cell cycle. A–B. Representative m^{6}A site at *chr5:69174992 (+ strand, CCNB1)* illustrating dynamic transitions in methylation levels. m^{6}A states are defined based on pre-splicing and post-splicing odds ratios (OR): sites with both pre-splicing OR < 0 and post-splicing OR < 0 are classified as *low* state; sites with both OR > 0 are classified as *high* state. Sites with pre-splicing OR > 0 and post-splicing OR < 0 are defined as *up* state, whereas sites with post-splicing OR > 0 and pre-splicing OR < 0 are defined as *down* state. **C.** Phase portrait of global m^{6}A dynamics across the cell cycle, aggregated over 142 sites with Gaussian smoothing applied. **D.** Phase portrait of global m^{6}A dynamics across the cell cycle, with phase labels assigned according to the state definitions described in panel (B).

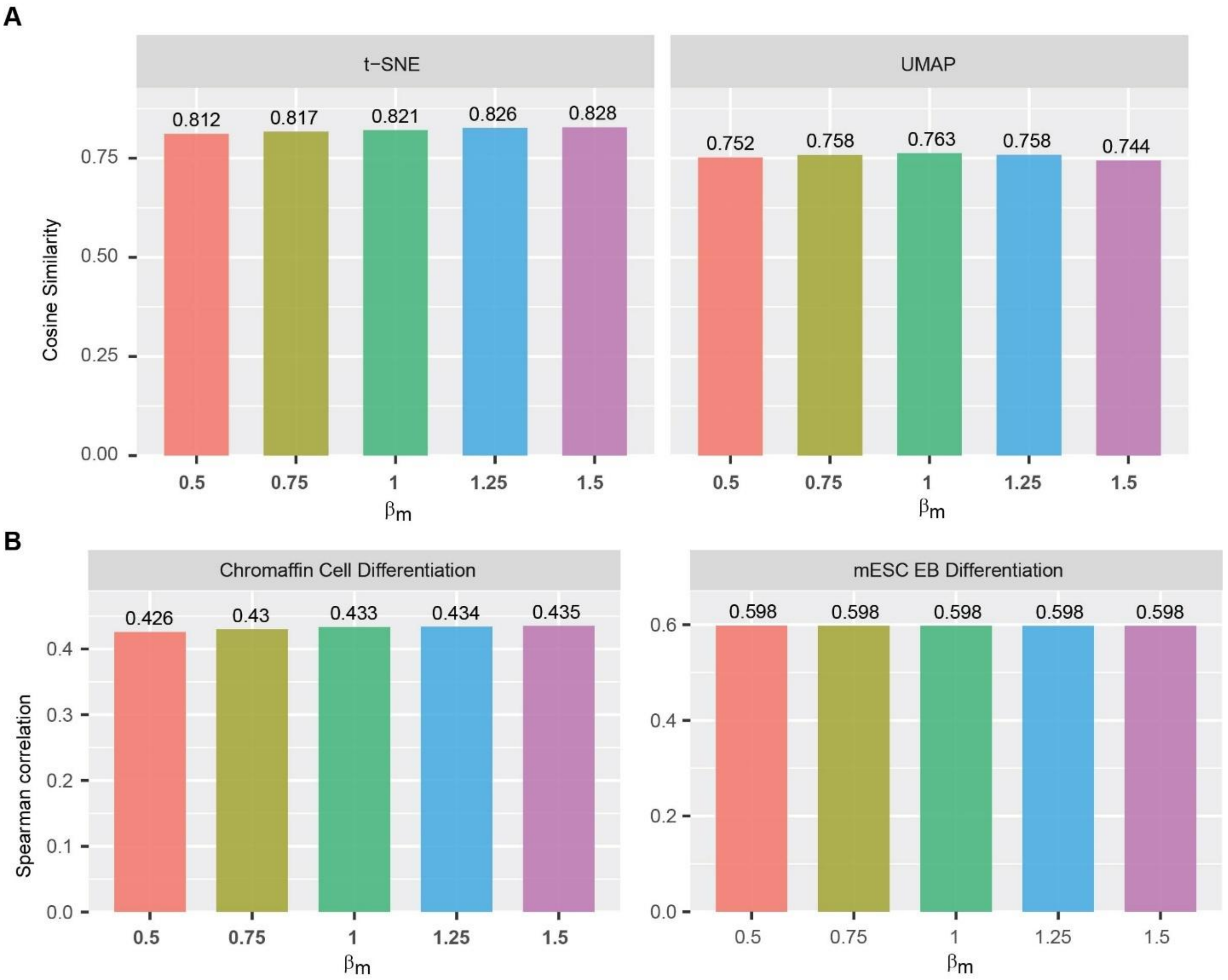

Supplementary Figure 13. Robustness of VeloRM trajectory inference to β_m perturbation. A. In silico perturbation analysis in the scDART-seq dataset using t-SNE and UMAP embeddings. The modified-RNA splicing rate β_m was varied from 0.5 to 1.5 while keeping the non-modified splicing rate unchanged. Performance was evaluated by cosine similarity between the predicted transition direction and the expected direction toward target cell states. **B.** In silico perturbation analysis in two scRNA-seq A-to-I editing datasets, including chromaffin cell differentiation and mESC embryoid body differentiation. Performance was evaluated by the Spearman correlation between inferred pseudotime and annotated differentiation states. The results remained stable across β_m values, indicating that VeloRM is robust to moderate βm misspecification.

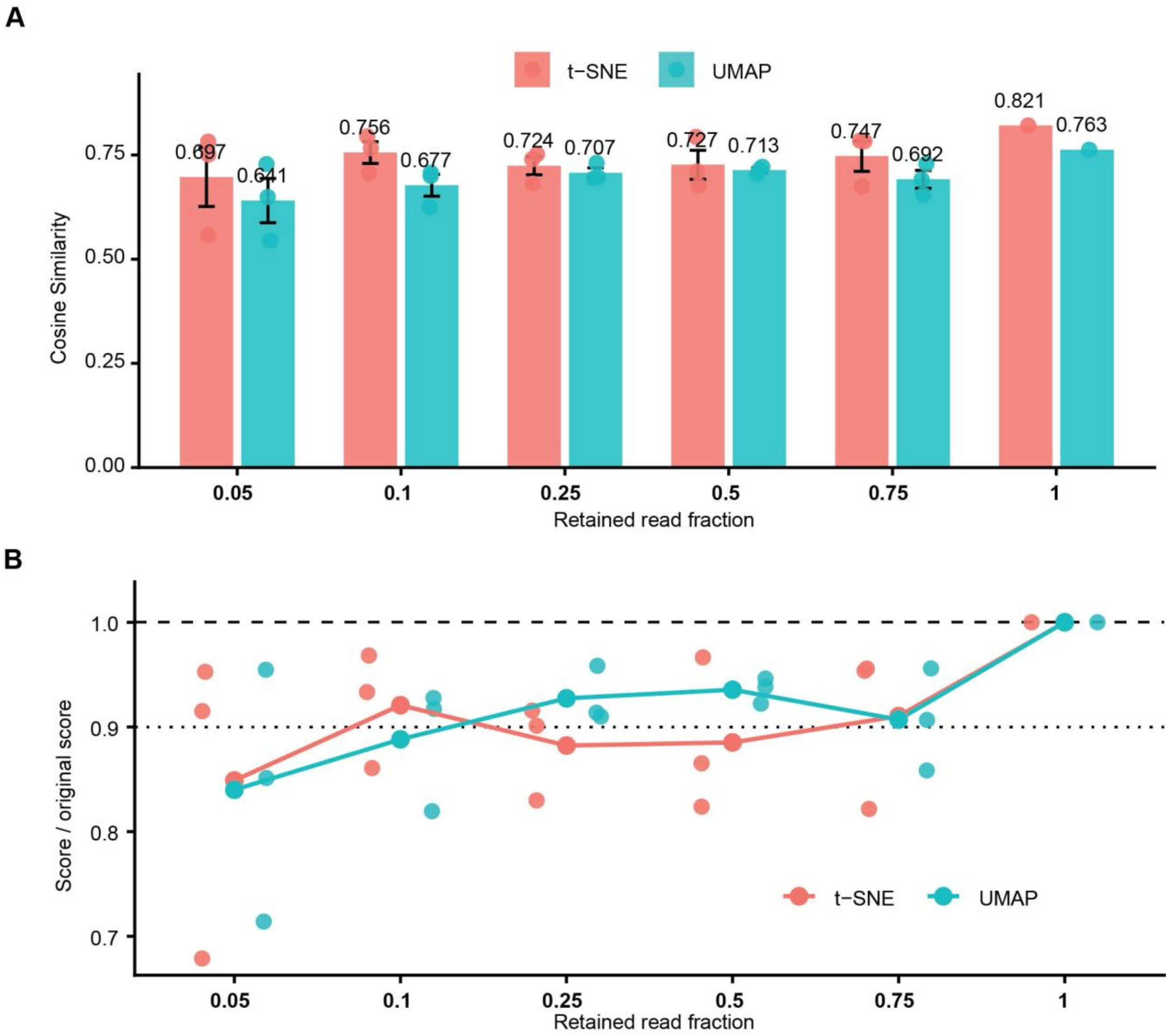

Supplementary Figure 14. Processed-matrix-level subsampling analysis of VeloRM trajectory recovery in the scDART-seq dataset. A. Mean cosine similarity after subsampling the retained spliced and unspliced modified-read matrices to 5%, 10%, 25%, 50%, and 75% of the retained signal, with three replicates for each fraction. The original full retained matrix is shown as fraction 1. Trajectory recovery was evaluated by the mean absolute cosine similarity between the predicted transition direction, derived from the transition probability matrix, and the expected direction toward target cell states. **B.** Relative trajectory recovery compared with the original full retained matrix, shown as the ratio of each subsampled score to the corresponding original score. Results are shown for both t-SNE and UMAP embeddings. VeloRM retained relatively stable trajectory recovery across moderate subsampling levels, although performance decreased at the lowest retained fractions.

REFERENCES

1. Williams, C.K. (1998), *Learning in graphical models*. Springer, pp. 599-621.
2. Anders, S., Huber, W. (2010) Differential expression analysis for sequence count data. *Nature Precedings*, 1-1.
3. Du, P., Zhang, X., Huang, C.-C. *et al.* (2010) Comparison of Beta-value and M-value methods for quantifying methylation levels by microarray analysis. *BMC bioinformatics*, **11**, 1-9.
4. Wang, Y., Chen, K., Wei, Z. *et al.* (2021) MetaTX: deciphering the distribution of mRNA-related features in the presence of isoform ambiguity, with applications in epitranscriptome analysis. *Bioinformatics*, **37**, 1285-1291.

5. Ke, S., Pandya-Jones, A., Saito, Y. *et al.* (2017) m6A mRNA modifications are deposited in nascent pre-mRNA and are not required for splicing but do specify cytoplasmic turnover. *Genes & development*, **31**, 990-1006.
6. Geula, S., Moshitch-Moshkovitz, S., Dominissini, D. *et al.* (2015) m6A mRNA methylation facilitates resolution of naïve pluripotency toward differentiation. *Science*, **347**, 1002-1006.
7. Zhou, K.I., Shi, H., Lyu, R. *et al.* (2019) Regulation of co-transcriptional pre-mRNA splicing by m6A through the low-complexity protein hnRNPG. *Molecular cell*, **76**, 70-81. e79.
8. Liu, N., Dai, Q., Zheng, G. *et al.* (2015) N 6-methyladenosine-dependent RNA structural switches regulate RNA–protein interactions. *Nature*, **518**, 560-564.
9. Song, S., Fan, G., Li, Q. *et al.* (2021) IDH2 contributes to tumorigenesis and poor prognosis by regulating m6A RNA methylation in multiple myeloma. *Oncogene*, **40**, 5393-5402.
10. Alharbi, A.B., Schmitz, U., Bailey, C.G., Rasko, J.E. (2021) CTCF as a regulator of alternative splicing: new tricks for an old player. *Nucleic acids research*, **49**, 7825-7838.
11. Liu, F., Zhou, Y. (2025) m6A-induced ribosome stalling and collision trigger mRNA decay. *Molecular Cell*, **85**, 2630-2632.
12. Zhou, Y., Ćorović, M., Hoch-Kraft, P. *et al.* (2024) m6A sites in the coding region trigger translation-dependent mRNA decay. *Molecular Cell*, **84**, 4576-4593. e4512.
13. Murakami, S., Olarerin-George, A.O., Liu, J.F. *et al.* (2025) m6A alters ribosome dynamics to initiate mRNA degradation. *Cell*.
14. Tang, P., Yang, J., Chen, Z. *et al.* (2024) Nuclear retention coupled with sequential polyadenylation dictates post-transcriptional m6A modification in the nucleus. *Molecular Cell*, **84**, 3758-3774. e3710.
15. Liu, N., Zhou, K.I., Parisien, M. *et al.* (2017) N 6-methyladenosine alters RNA structure to regulate binding of a low-complexity protein. *Nucleic acids research*, **45**, 6051-6063.
16. Wang, X., Lu, Z., Gomez, A. *et al.* (2014) N 6-methyladenosine-dependent regulation of messenger RNA stability. *Nature*, **505**, 117-120.
17. Jones, C.I., Zabolotskaya, M.V., Newbury, S.F. (2012) The 5′ → 3′ exoribonuclease XRN1/Pacman and its functions in cellular processes and development. *Wiley Interdisciplinary Reviews: RNA*, **3**, 455-468.
18. Mauer, J., Luo, X., Blanjoie, A. *et al.* (2017) Reversible methylation of m6Am in the 5′ cap controls mRNA stability. *Nature*, **541**, 371-375.
19. Dominissini, D., Moshitch-Moshkovitz, S., Schwartz, S. *et al.* (2012) Topology of the human and mouse m6A RNA methylomes revealed by m6A-seq. *Nature*, **485**, 201-206.
20. Tan, M.H., Li, Q., Shanmugam, R. *et al.* (2017) Dynamic landscape and regulation of RNA editing in mammals. *Nature*, **550**, 249-254.
21. Hsiao, Y.-H.E., Bahn, J.H., Yang, Y. *et al.* (2018) RNA editing in nascent RNA affects pre-mRNA splicing. *Genome research*, **28**, 812-823.
22. Rieder, L.E., Savva, Y.A., Reyna, M.A. *et al.* (2015) Dynamic response of RNA editing to temperature in Drosophila. *BMC biology*, **13**, 1-16.
23. Levanon, E.Y., Eisenberg, E., Yelin, R. *et al.* (2004) Systematic identification of abundant A-to-I editing sites in the human transcriptome. *Nature biotechnology*, **22**, 1001-1005.
24. Eggington, J.M., Greene, T., Bass, B.L. (2011) Predicting sites of ADAR editing in double-stranded RNA. *Nature communications*, **2**, 319.
25. Pinto, Y., Buchumenski, I., Levanon, E.Y., Eisenberg, E. (2018) Human cancer tissues exhibit reduced A-to-I editing of miRNAs coupled with elevated editing of their targets. *Nucleic acids research*, **46**, 71-82.
26. Hundley, H.A., Krauchuk, A.A., Bass, B.L. (2008) C. elegans and H. sapiens mRNAs with edited 3′ UTRs are present on polysomes. *Rna*, **14**, 2050-2060.
27. Desterro, J.M., Keegan, L.P., Lafarga, M. *et al.* (2003) Dynamic association of RNA-editing enzymes with the nucleolus. *Journal of cell science*, **116**, 1805-1818.
28. Meyer, K.D., Saletore, Y., Zumbo, P. *et al.* (2012) Comprehensive analysis of mRNA methylation reveals enrichment in 3' UTRs and near stop codons. *Cell*, **149**, 1635-1646.
29. Dobin, A., Davis, C.A., Schlesinger, F. *et al.* (2013) STAR: ultrafast universal RNA-seq aligner. *Bioinformatics*, **29**, 15-21.
30. Love, M.I., Huber, W., Anders, S. (2014) Moderated estimation of fold change and dispersion for RNA-seq data with DESeq2. *Genome biology*, **15**, 1-21.

31. La Manno, G., Soldatov, R., Zeisel, A. *et al.* (2018) RNA velocity of single cells. *Nature*, **560**, 494-498.
32. Linder, B., Sharma, P., Wu, J. *et al.* (2025) tRNA modifications tune m6A-dependent mRNA decay. *Cell*.
33. Zaccara, S., Ries, R.J., Jaffrey, S.R. (2019) Reading, writing and erasing mRNA methylation. *Nature reviews Molecular cell biology*, **20**, 608-624.
34. Louloupi, A., Ntini, E., Conrad, T., Ørom, U.A.V. (2018) Transient N-6-methyladenosine transcriptome sequencing reveals a regulatory role of m6A in splicing efficiency. *Cell reports*, **23**, 3429-3437.
